# CDK1 serves as a therapeutic target of adrenocortical carcinoma via regulating epithelial–mesenchymal transition, G2/M phase transition, and PANoptosis

**DOI:** 10.1186/s12967-022-03641-y

**Published:** 2022-10-02

**Authors:** Liwen Ren, Yihui Yang, Wan Li, Xiangjin Zheng, Jinyi Liu, Sha Li, Hong Yang, Yizhi Zhang, Binbin Ge, Sen Zhang, Weiqi Fu, Dexin Dong, Guanhua Du, Jinhua Wang

**Affiliations:** 1 The State Key Laboratory of Bioactive Substance and Function of Natural Medicines, Beijing, 100050 China; 2 Key Laboratory of Drug Target Research and Drug Screen, Institute of Materia Medica, Chinese Academy of Medical Science and Peking Union Medical College, Beijing, 100050 China; 3 Department of Urology, Peking Union Medical College Hospital, Beijing, 100730 China

**Keywords:** Adrenocortical carcinoma, CDK1, Cucurbitacin E, Mitotane, EMT, Epithelial-mesenchymal transition, PANoptosis

## Abstract

**Background:**

Adrenocortical carcinoma (ACC) is an extremely rare, aggressive tumor with few effective therapeutic options or drugs. Mitotane (Mtn), which is the only authorized therapeutic drug, came out in 1970 and is still the only first-line treatment for ACC in spite of serious adverse reaction and a high recurrence rate.

**Methods:**

By in silico analysis of the ACC dataset in the cancer genome atlas (TCGA), we determined that high expression levels of cyclin-dependent kinase-1 (CDK1) were significantly related to the adverse clinical outcomes of ACC. In vitro and in vivo experiments were performed to evaluate the role of CDK1 in ACC progression through gain and loss of function assays in ACC cells. CDK1 inhibitors were screened to identify potential candidates for the treatment of ACC. RNA sequencing, co-immunoprecipitation, and immunofluorescence assays were used to elucidate the mechanism.

**Results:**

Overexpression of CDK1 in ACC cell lines promoted proliferation and induced the epithelial-to-mesenchymal transition (EMT), whereas knockdown of CDK1 expression inhibited growth of ACC cell lines. The CDK1 inhibitor, cucurbitacin E (CurE), had the best inhibitory effect with good time-and dose-dependent activity both in vitro and in vivo. CurE had a greater inhibitory effect on ACC xenografts in nude mice than mitotane, without obvious adverse effects. Most importantly, combined treatment with CurE and mitotane almost totally eliminated ACC tumors. With respect to mechanism, CDK1 facilitated the EMT of ACC cells via Slug and Twist and locked ACC cells into the G2/M checkpoint through interaction with UBE2C and AURKA/B. CDK1 also regulated pyroptosis, apoptosis, and necroptosis (PANoptosis) of ACC cells through binding with the PANoptosome in a ZBP1-dependent way.

**Conclusions:**

CDK1 could be exploited as an essential therapeutic target of ACC via regulating the EMT, the G2/M checkpoint, and PANoptosis. Thus, CurE may be a potential candidate drug for ACC therapy with good safety and efficacy, which will meet the great need of patients with ACC.

**Supplementary Information:**

The online version contains supplementary material available at 10.1186/s12967-022-03641-y.

## Background

Adrenocortical carcinoma (ACC) is an extremely infrequent neoplasm and makes up only a small percentage of adrenal tumors that are initiated in the adrenal cortex [1]. The neoplasm has high heterogeneity with poor prognosis and lacks effective pharmaceutical treatment options [2]. Apart from radical surgery, there are few therapeutic options. Mitotane (Mtn), which is the only authorized drug for treatment, appeared in 1970 after approval by the Food and Drug Administration (FDA), and is still the only first-line treatment for ACC [3]. Because of endocrine metabolic disturbances and metastatic tumor growth, ACC patients frequently suffer serious illness [4]. Therefore, exploring novel targets and treatments for patients with ACC is urgently necessary.

By analyzing the survival-related genes of ACC patients, we found that the expression of cyclin-dependent kinase-1 (CDK1) was significantly correlated with the survival of ACC patients and is likely to be a therapeutic target for ACC. CDK1 is involved in the switching between the G2 phase and cell mitosis and enhanced CDK1 activity is often seen in neoplastic cells. Therefore, CDK1 was recognized as a likely target for the treatment of cancer [5]. Several CDK1 inhibitors such as Rigosertib (phase II/III) [6] and Zotiraciclib have entered the phase I clinical research stage for the treatment of pancreatic cancer and glioma [7]. Inhibition of CDK1 has been related to effective therapeutic outcomes for breast cancer therapy whether used alone or in combination with other drugs [8]. CDK1 could elevate the development of melanoma via interacting with Sox2 [9]. Despite the rarity of the disease, there is an urgent need to develop safe and effective therapeutic drugs for ACC. CDK1 is a classic therapeutic target and with many advantages for the rapid development of ACC drug candidates. Unfortunately, the specific roles and mechanisms of CDK1 in ACC are unclear, and there are still many unknowns about the therapeutic and prognostic significance of CDK1 in ACC that require investigation.

The epithelial-mesenchymal transition (EMT) is the original process in tumor metastasis in which epithelial cells assume mesenchymal characteristics [10]. The EMT is closely related to tumor initiation, metastasis, invasion and drug resistance, which plays an important role in cancer progression [11]. The expression of E-cadherin is downregulated and the expression of N-cadherin and metalloproteinases (MMPs) are increased when the EMT process is activated. The EMT is regulated by several transcription factors, such as those of the SNAIL and TWIST family [12]. However, there have been few studies on the relationship between CDK1 and tumor metastasis or EMT.

Programmed cell death (PCD), which is mediated via an evolutionarily conserved signaling pathway, plays an essential part in antitumor drug discovery [13]. A novel pathway identified for PCD, called PANoptosis, is mediated through a PANoptosome which is a newly recognized multimeric cytoplasmic protein complex [14]. PANoptosis is a type of synchronized cell death involving co-regulation and crosstalk among pyroptosis, apoptosis, and necroptosis [15]. The PANoptosome involves three crucial effectors of PCD PANoptosis operating in parallel. Z-DNA binding protein-1 (ZBP1) activates inflammatory cell death and PANoptosis and is an essential regulator in the formation of the PANoptosome [16]. So far, there has been no relevant research on PANoptosis in ACC or its relationship with CDK1.

In our research, it was discovered that abnormally elevated CDK1 expression was strongly related to the unfavorable prognosis of ACC patients. In vitro and in vivo experiments showed that overexpression of CDK1 promoted proliferation and EMT of ACC cell lines whereas knockdown of CDK1 expression inhibited these functions in ACC cell lines. Through screening of CDK1 inhibitors in ACC cell lines, it was identified that cucurbitacin E (CurE) had the best inhibitory effect with good time-and dose-dependent responses both in vitro and in vivo. In addition, CurE was better at inhibiting ACC tumors in a nude mouse xenograft model than mitotane. Whether used alone or in combination with mitotane, CurE showed no obvious adverse effects. CDK1 facilitated the EMT of ACC cells via Slug and Twist and regulated the G2/M phase transition of ACC cells through interactions with UBE2C and AURKA/B. CDK1 regulated the apoptosis, pyroptosis, and necroptosis (PANoptosis) of ACC cells through binding with the PANoptosome in a ZBP1-dependent way. Taken together, the above results indicate that CDK1 could serve as an effective antitumor target for ACC with prognostic and therapeutic value, and CurE is a likely candidate with good safety and efficacy for the treatment of ACC.

## Methods

### Bioinformatics exploration

CDK1 expression in normal and tumor group was investigated in the following databases: http://gepia.cancer-pku.cn/ [17], http://gdac.broadinstitute.org/ [18] and https://www.oncomine.org/ [19]. The mRNA level of CDK1 in ACC was analyzed on the website, http://gepia.cancer-pku.cn/, http://ualcan.path.uab.edu/index.html [20]. The prognostic value of CDK1 for ACC was analyzed on the website, http://sangerbox.com/Index.

### Cell culture

The human ACC cell lines, NCI-H295R and SW-13, were acquired from Procell Life Science &Technology (Wuhan, China). SW-13 cells were maintained in Leibovitz's L-15, with 10% FBS and 1% P/S (Procell, CM-0451). NCI-H295R cells were maintained in DMEM/F12, with 6.25 μg/mL insulin, 6.25 ng/mL selenium, 6.25 μg/mL transferrin, 5.35 μg/mL linoleic acid, 10% FBS and 1% P/S (Procell, CM-0399).

### Stable-cell line establishment via lentiviral transfection

For stable CDK1 overexpression in SW-13 cells (SW13_CDK1), the pSLenti-SFH-EGFP-P2A-Puro-CMV-CDK1-3xFLAG-WPRE and corresponding control lentivirus were obtained from Hanbio (Shanghai, China). After transfection into SW-13 for 72 h, 2 μg/mL puromycin was added to the culture medium screen for stable cloning. Two shRNAs were used to construct a stable CDK1-knockdown NCI-H295R cell line (NCI-H295R_shCDK1). The sequences of the two shRNAs were as follows: 5′-GTGGAATCTTTACAGGACTAT-3′ and 5′-GATTCAGAAATTGATCAACTC-3′. Puromycin (2 μg/mL) was added to cultures to obtain stable CDK1 knockdown in NCI-H295R cells.

### CDK1 inhibitors and other chemical reagents

The inhibitors used in the pharmacological activity test for ACC were obtained from MedChemExpress (New Jersey, USA). The CDK1 inhibitors were prazosin hydrochloride, HY-B0193A, 19237-84-4; cucurbitacin E (CurE), HY-N0417, 18444-66-1; Ro-3306, HY-12529, 872573-93-8; and AZD-5438, HY-10012, 602306-29-6; the GSK3 inhibitors were IX, HY-10580, 667463-62-9 and mitotane, HY-13690, 53-19-0; the pan-caspase inhibitor was Z-VAD-FMK (VAD), HY-16658B, 161401-82-7; the inhibitor of autophagy was 3-methyladenine (3-MA), HY-19312, 5142-23-4; and the necroptosis inhibitor was necrosulfonamide (Nec), HY-100573, 1360614-48-7. Additional reagents were cisplatin (Pt), HY-17394, 15663–27-1; paclitaxel (Ptx), HY-B0015, 33069-62-4; and cycloheximide (CHX), HY-12320, 66-81-9.

### In vitro cell viability assay

Cell proliferation was measured by CCK8 kit (Dojindo, Kumanmoto, Japan). SW-13_CDK1, NCI-H295R_shCDK1 and corresponding control cells were seeded in 96-well plates at 3 × 10^3^ cells per well and cultured for 24, 48 and 72 h. Subsequently, CCK8 reagent was added (10 μL/well) and after incubation for 1 h, the absorbance at 450 nm was measured. To calculate the IC_50_ of CDK1 inhibitors, cells were cultured in 96-well plates with 3 × 10^3^ cells per well at 37 °C with different concentration of the CDK1 inhibitor, CurE (0–10 μmol/L). After culturing for 24, 48 and 72 h, 10 μL of CCK8 reagent was added per well. After incubation for 1 h, the absorbance at 450 nm was measured.

NCI-H295R and SW-13 cells were pretreated with different inhibitors including Z-VAD-FMK (50 μmol/L), 3-MA (2 mmol/L), and necrosulfonamide (2 μmol/L) for 2 h. Afterwards, the SW-13 cells were incubated with 0.1 μmol/L CurE and NCI-H295R cells were treated with 1 μmol/L CurE for 24 h. The cell viability was measured according to the above method.

### DNA synthesis assay

The EdU kit was used according to the manufacture’s protocol. SW-13_CDK1, NCI-H295R_shCDK1, and the corresponding control cells were cultured for 24 h. Afterwards, the EdU reagent (50 μmol/L final) was added to each and incubation at 37 °C was continued for another 24 h, after which the cells were fixed, permeabilized and stained with Hoechst 33342 and Apollo 567.

### Cell membrane integrity assessed by LDH release

A lactate dehydrogenase (LDH) detection kit (Dojindo, Japan) was used to measure the level of LDH released from cells. Cells were seeded at 3 × 10^3^/well in 96-well plates and treated with CurE for 24 h and 48 h. After incubation with CurE, the medium was discarded and 100 μL of working reagent was added to each well. Thirty minutes later, the reaction was ended with the stop solution and the activity was measured at 490 nm.

### Invasion and migration assay

The roles of CDK1 and CurE in invasion and migration of NCI-H295R and SW-13 cells were evaluated using a 6.5 mm transwell (#3422, Corning Costar, NY, USA). For the migration assay, 2 × 10^4^ cells were put into the upper chamber. For the invasion assay, the transwell chamber was coated with Matrigel dissolved in Biocoat (Corning USA). The Matrigel was diluted with FBS-free medium at a ratio of 1:6 and then 1 × 10^5^ cells were added into the transwell chamber. After incubation, the cells that crossed the insert were fixed, stained with 1% crystal violet, photographed, and counted under a visible light microscope (Nikon Eclipse Ti-U, Tokyo, Japan).

### Annexin V-FITC assay with propidium iodide (PI) staining

Cell apoptosis was determined using the annexin V-FITC/PI detection kit (Solarbio, Beijing, China). SW-13 cells were incubated with 0, 0.03, 0.1 and 0.3 μmol/L CurE and NCI-H295R cells with 0, 0.3, 1 and 3 μmol/L CurE for 24 and 48 h. After incubation, the cells were harvested and stained with annexin V-FITC and PI for 10 min. Flow cytometry (FACSVERSE, BD Biosciences, New Jersey, USA) was used to determine the percentage of early apoptotic cells, late apoptotic cells, pyroptotic cells and necrotic cells. The data on apoptosis rate was quantified by FlowJo software (v10). ACC cells were pretreated with necrosulfonamide (2 μmol/L) for 2 h and then treated with CurE for 24 h. The percentages of early apoptotic, late apoptotic, pyroptotic and necrotic cells were calculated using the above method.

### Cell cycle analysis

SW-13 cells were incubated with 0, 0.03, 0.1 and 0.3 μmol/L CurE and NCI-H295R cells with 0, 0.3, 1 and 3 μmol/L CurE for 24 and 48 h. Then cells were harvested by centrifugation and fixed in 75% ethanol for 24 h at 4 °C. After centrifugation, cells were stained with PI for 20 min, fluorescence was measured, and data was processed with FlowJo software (v10).

### RNA sequencing

SW-13_CDK1 cells, SW-13 cells administrated with 0.1 μmol/L CurE for 24 h and corresponding control cells were collected in TRIzol reagent. The next generation sequencing of the prepared isolates was conducted on an Illumina sequencer in Novogene Corporation (Beijing, China). The integrity and concentration of total RNA were qualified by the Agilent 2100 bioanalyzer and NanoDrop spectrophotometer. Illumina sequencer was used to build and qualify, then pool and sequence for the establishment of the New England Biolabs (NEB) libraries. The Gene ontology (GO) analysis were conducted on the DAVID database (v6.8) (https://david.ncifcrf.gov/).

### Knockdown of Slug, Twist and ZBP1 expression

To knock down Slug, Twist and ZBP1 expression, 100 nmol/L relative target siRNA and relative control siRNA (Genechem, Suzhou, China) were transfected into NCI-H295R and SW-13 cells using Lipofectamine 3000 reagent and cultured for 48 h at 37 °C. After transfection, SW-13 cells were incubated with 0.1 μmol/L CurE and NCI-H295R cells were incubated with 1 μmol/L CurE for 24 h.

### Co-immunoprecipitation (Co-IP)

SW-13_CDK1 and SW-13_NC cells were planted in 100 mm dishes, then collected in 500 μL lysis buffer on ice for 30 min. The lysates were centrifugated for 15 min (4 ℃, 12,000 *g*) and supernatant was collected. Samples was added with 10 μL DDDDK-Agarose (bimake, Shanghai, China) and rotated overnight at 4 ℃. The beads were washed, 20 μL of 2 × SDS-PAGE loading buffer was added to each, and heated at 100 °C for 5 min for Western blotting analysis.

The protein lysates of control and CurE treated cells were obtained as described. Equal quantities of proteins were incubated with CDK1 antibodies or IgG control and rotated overnight at 4 ℃. Protein complexes were collected with protein A/G agarose beads (Beyotime Technology) followed by washing 5 times and prepared for Western blotting.

### Western blotting

RIPA reagent with protease and phosphatase inhibitor cocktail was used to extract total protein (Applygen, Beijing, China) for 30 min on ice. The lysates were centrifuged at 12,000 *g* for 15 min at 4 °C and supernatants were collected. Equivalent amounts of protein were separated by SDS-PAGE, transferred to PVDF membranes, and incubated with primary antibodies (Additional file 8: Table S1) at 4 °C for 12 h. The blots were then washed and incubated with HRP-conjugated secondary antibodies. After washing, the blots were incubated with SuperECL reagent, protein bands were visualized, and image densities were quantitated using a Tanon imaging system.

### Xenograft model of nude mice

All animal experiments were carried out following the NIH Guide for the Care and Use of Laboratory Animals. Female BALB/c-nu nude mice (16–18 g) were obtained from the Charles River Co. (Beijing, China) and housed at the Institute of Materia Medica animal barrier facility.

In the first part, mice were randomly divided into six per group, and aliquots of 1 × 10^6^ SW-13_NC, SW-13_CDK1, NCI-H295R_shNC, or NCI-H295R_shCDK1 cells were injected subcutaneously into the right flank (n = 6). The tumor volume (mm^3^) and body weight (g) were measured every 2 days. At the end of experiment, the mice were euthanized, and the tumors were excised, weighed and photographed.

To determine the effect of CDK1 inhibitor treatment, SW-13 cells (1 × 10^8 ^cells/mL) were subcutaneously injected into the right sides of the mice. After 14 days, when the tumor volume had reached about 100 mm^3^, the mice were randomly assigned to five groups (n = 6) for the following treatments: (1) no treatment, (2) 40 mg/kg mitotane i.p., (3) 10 mg/kg CurE i.p., (4) 20 mg/kg CurE, i.p., and (5) 10 mg/kg CurE plus 40 mg/kg mitotane, i.p., once a day for 3 weeks. Body weight (g) and tumor volume (V, mm^3^) were measured every 2 days. All the mice were euthanized after 3 weeks, and the organs and tumors were collected, weighed and photographed.

### Immunohistochemistry (IHC) and immunofluorescence (IF)

Immunohistochemistry (IHC) and immunofluorescence (IF) were carried out using kits according to manufacturer’s instructions. The expression of Slug and Twist was detected via IHC staining in the tumor tissues formed from SW-13_NC, SW-13_CDK1, NCI-H295R_shNC, NCI-H295R_shCDK1 cells in the xenograft assays. The expression of ZBP1 was detected in tumor tissues from mice treated with 10 mg/kg CurE (low) and 20 mg/kg CurE (high) via IF staining. A Nikon Eclipse Ti microscope was used to image the stained sections.

### Statistical analysis

All the results are shown as mean ± standard deviation (SD). Statistical significance among the groups was determined using GraphPad Prism 7.0 with one-way ANOVA, and *P* < 0.05 was considered statistically significant.

## Results

### CDK1 could be a therapeutic target and prognostic biomarker for ACC

To investigate the expression pattern of CDK1 in ACC, bioinformatic analysis was conducted on the GTEx, TCGA, and Oncomine databases. Results suggested that CDK1 was abnormally elevated in the majority of tumors relative to healthy controls (Additional file 1: Fig. S1 a–b). CDK1 was abnormally elevated in ACC (Additional file 1: Fig. S1c) and the elevated expression of CDK1 closely tracked with the pathological stage (Additional file 1: Fig. S1d) and nodal metastatic status of ACC patients (Additional file 1: Fig. S1e). Correlation analysis results also indicated that CDK1 expression was related to TNM stage (*P* < 0.001), tumor status (*P* < 0.001) and pathological stage (*P* < 0.001). In addition, the expression level of CDK1 was associated with the outcome of primary therapy (*P* < 0.001), overall survival (OS) (*P* < 0.001), disease-specific survival (DSS) (*P* < 0.001) and progression-free interval (PFI) (*P* < 0.001) (Table 1). The Kaplan-Meier analysis also indicated a relationship between CDK1 expression and OS (*P* < 0.0001), DSS (*P* < 0.0001) and PFI (*P* < 0.0001) survival probability of ACC patients (Additional file 1: Fig. S1f–h). ROC curve of CDK1 expression and OS, DSS and PFI survival suggested that CDK1 could be used as a prognostic indicator of ACC (Additional file 1: Fig. S1i–k). The above results indicated that CDK1 was abnormally elevated in ACC and that its expression level could serve as a prognostic biomarker as well as a potential therapeutic target for ACC.

**Table 1:** Correlation analysis results between CDK1 expression and prognostic indicators

Characteristic	Level	Low expression of CDK1	High expression of CDK1	*P* value
n		39	40	
T stage, n (%)	T1	7 (9.1%)	2 (2.6%)	< 0.001
	T2	28 (36.4%)	14 (18.2%)	
	T3	2 (2.6%)	6 (7.8%)	
	T4	2 (2.6%)	16 (20.8%)	
N stage, n (%)	N0	36 (46.8%)	32 (41.6%)	0.310
	N1	3 (3.9%)	6 (7.8%)	
M stage, n (%)	M0	36 (46.8%)	26 (33.8%)	0.018
	M1	3 (3.9%)	12 (15.6%)	
Pathologic stage, n (%)	Stage I	7 (9.1%)	2 (2.6%)	< 0.001
	Stage II	25 (32.5%)	12 (15.6%)	
	Stage III	4 (5.2%)	12 (15.6%)	
	Stage IV	3 (3.9%)	12 (15.6%)	
Tumor status, n (%)	Tumor free	29 (37.7%)	10 (13%)	< 0.001
	With tumor	10 (13%)	28 (36.4%)	
Primary therapy outcome, n (%)	PD	3 (4.5%)	15 (22.4%)	< 0.001
	SD	2 (3%)	0 (0%)	
	PR	1 (1.5%)	0 (0%)	
	CR	32 (47.8%)	14 (20.9%)	
OS event, n (%)	Alive	35 (44.3%)	16 (20.3%)	< 0.001
	Dead	4 (5.1%)	24 (30.4%)	
DSS event, n (%)	Alive	35 (45.5%)	16 (20.8%)	< 0.001
	Dead	4 (5.2%)	22 (28.6%)	
PFI event, n (%)	Alive	27 (34.2%)	11 (13.9%)	< 0.001
	Dead	12 (15.2%)	29 (36.7%)	

### CDK1 facilitated the proliferation of ACC cells in vitro and in vivo

**Fig. 1:**
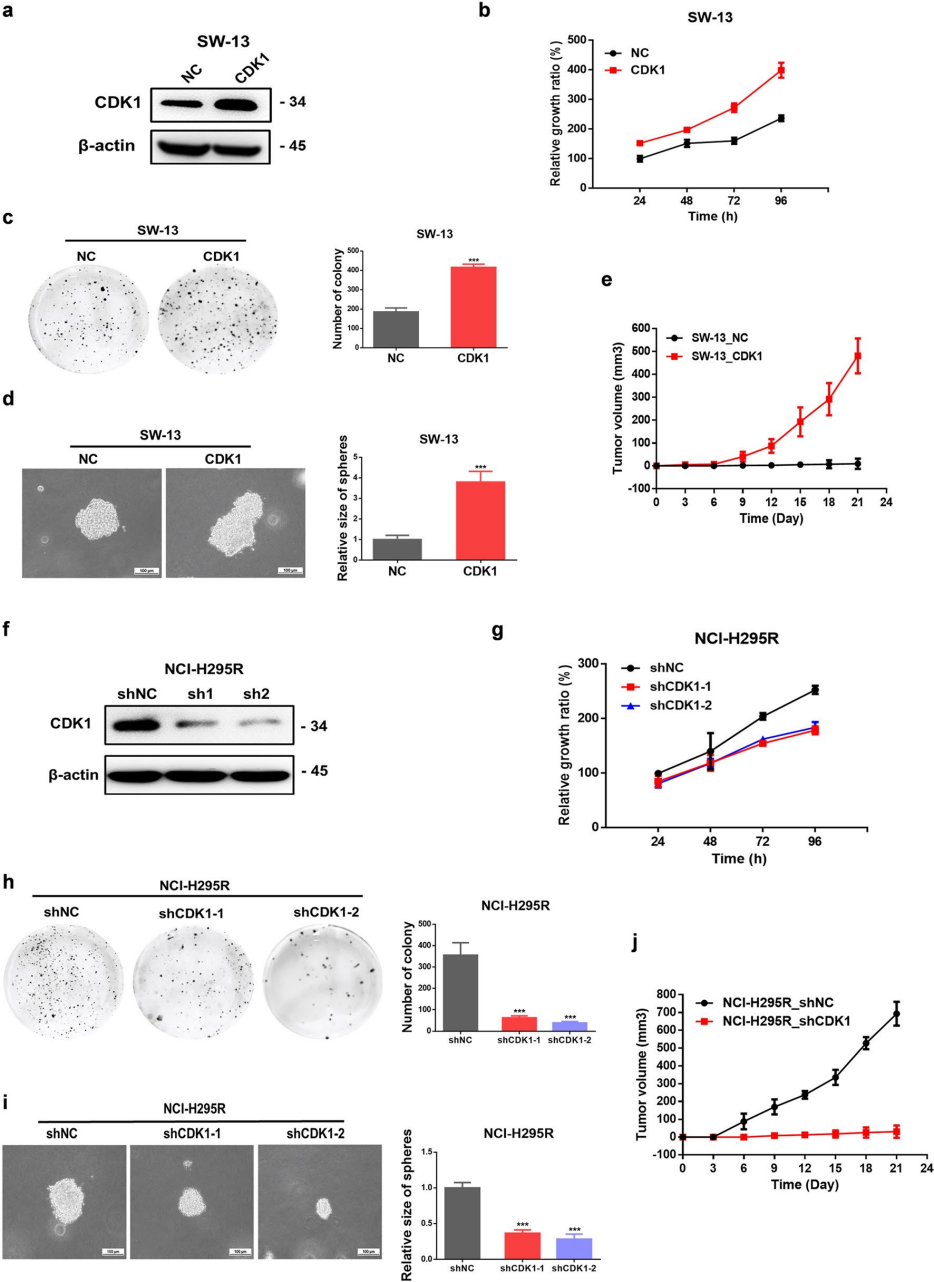
CDK1 facilitated the proliferation of ACC cells both in vitro and in vivo. **a** Transfection efficiency of CDK1 overexpression in SW-13 cells. **b** Upregulation of CDK1 promoted growth of SW-13 cells. **c** Overexpression of CDK1 increased the colony formation of SW-13 cells in soft agar. **d** Upregulation of CDK1 facilitated the proliferation of SW-13 cells in 3D Matrigel. **e** Upregulation of CDK1 facilitated the proliferation of SW-13 cells in vivo. **f** Transfection efficiency of knockdown of CDK1 expression in NCI-H295R cells. **g** Downregulation of CDK1 suppressed growth of NCI-H295R cells. **h** Knockdown of CDK1 expression suppressed soft agar colony formation of NCI-H295R cells. **i** Downregulation of CDK1 suppressed growth of NCI-H295R cells in 3D Matrigel. **j** Downregulation of CDK1 suppressed growth of NCI-H295R cells in vivo. Experiments were performed in triplicate, and data was presented as mean ± SD. ***P < 0.001 vs. control group

To assess the influence of CDK1 on the proliferation of ACC cells, the growth in liquid medium, 3D Matrigel, soft agar, and nude mice was evaluated. Upregulation of CDK1 increased the growth of SW-13 cells in culture medium (Fig. 1a–b), promoted colony formation in soft agar (Fig. 1c), invasiveness in 3D Matrigel (Fig. 1d), and the growth of tumors in nude mice (Fig. 1e). In contrast, downregulation of CDK1 in NCI-H295R cells remarkably suppressed their growth (Fig. 1f–g), colony formation in soft agar (Fig. 1h), and invasiveness in 3D Matrigel (Fig. 1i). Downregulating CDK1 in NCI-H295R cells also suppressed the growth of these tumor cells in nude mice (Fig. 1j). Thus, CDK1 is significantly associated with the proliferation of ACC cells and could be utilized as a potential target for ACC therapy.

### Differential gene expression and enrichment analysis after upregulation of CDK1 in SW-13 cells

The expression of CDK1 in ACC cell lines was determined by Western blotting, and the results showed that CDK1 expression was lower in SW-13 cells than in NCI-H295R cells (Additional file 2: Fig. S2a). To investigate the underlying mechanism of CDK1’s effects in ACC, the sequences of RNA isolated from cells in the SW-13_CDK1 group were compared to those from the SW-13_NC group. There were 66 significantly upregulated genes and 104 downregulated genes in the SW-13_CDK1 group (Additional file 2: Fig. S2b). The GO enrichment results of differentially-expressed genes (DEGs) suggested that CDK1 participated in cell-cell adhesion, muscle structure development, angiogenesis and cell proliferation. (Additional file 2: Fig. S2c). KEGG pathway enrichment of DEGs also indicated that CDK1 could regulate cell adhesion molecules, epithelial cell signaling, and cell cycle pathways (Additional file 2: Fig. S2d). Reactome enrichment indicated that CDK1 might play an essential role in GPCR signaling (Additional file 2: Fig. S2e). Based on these RNA-seq results, we speculated that CDK1 played a vital part in the proliferation and metastasis of ACC cells.

#### CDK1 facilitated the EMT of ACC cells

**Fig. 2:**
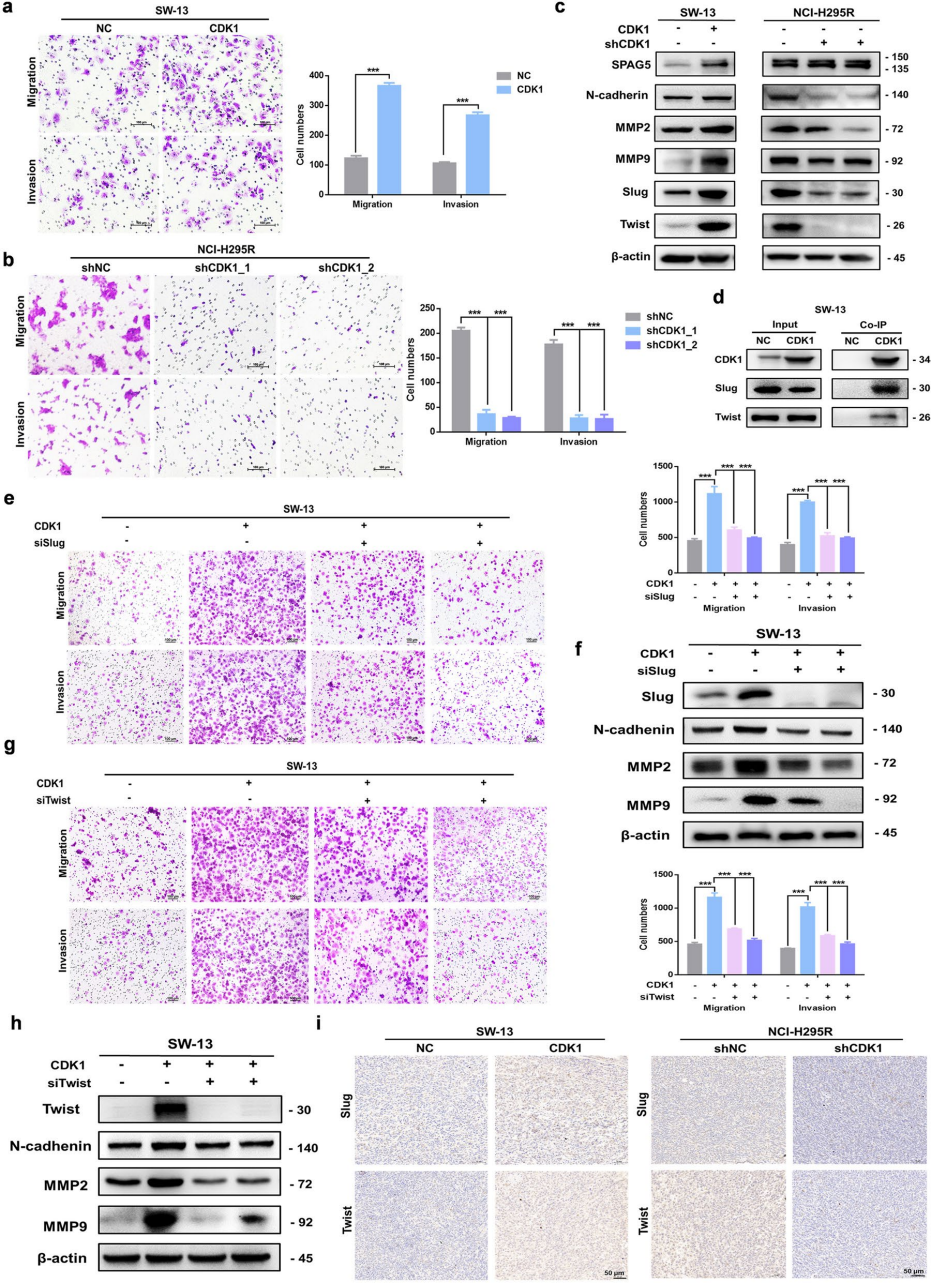
CDK1 facilitated the epithelial-mesenchymal transition of ACC cells via Slug and Twist. **a** Overexpression of CDK1 elevated invasion and migration of SW-13 cells. **b** Knockdown of CDK1 expression reduced migration and invasion of NCI-H295R cells. **c** CDK1 regulated expression of metastasis-related proteins. **d** CDK1 interacted with Slug and Twist. **e** Knockdown of Slug reversed the pro-metastatic effect of CDK1. **f** Effect of Slug on CDK1-regulated pathways of metastasis. **g** Knockdown of Twist blocked CDK1’s pro-metastatic activity. **h** Effect of Twist on CDK1-regulated pathways of metastasis. **i** CDK1 influenced expression of Slug and Twist in vivo. Experiments were performed in triplicate, and data was presented as mean ± SD. ***P < 0.001 vs. control group

To further evaluate the role of CDK1 on the metastasis of ACC cells, migration and invasion experiments were conducted. Results showed that upregulation of CDK1 elevated the invasion and migration capability of SW-13 cells (Fig. 2a), while downregulation of CDK1 suppressed the invasion and migration of NCI-H295R cells (Fig. 2b). CDK1 could also influence the EMT process of ACC cells by altering the expression of EMT-related essential proteins like SPAG5, N-cadherin, MMP2/7, Slug and Twist (Fig. 2c). Co-IP experiments were performed to investigate downstream targets of CDK1 and results were consistent with interaction between CDK1 and Slug and Twist, which are essential proteins in metastatic signaling (Fig. 2d). Knockdown of Slug (Fig. 2e) or Twist (Fig. 2g) reversed the pro-metastatic effect of CDK1 and knockdown of Slug (Fig. 2f) or Twist (Fig. 2h) could inhibit CDK1-regulated pathways of metastasis. IHC experiments suggested that CDK1 also influenced the expression of Slug and Twist in vivo (Fig. 2i). Thus, we conclude that CDK1 could facilitate the EMT in ACC cells by regulating Slug and Twist.

### CDK1 inhibitor CurE reduced the proliferation of ACC cells in vitro and in vivo

**Fig. 3:**
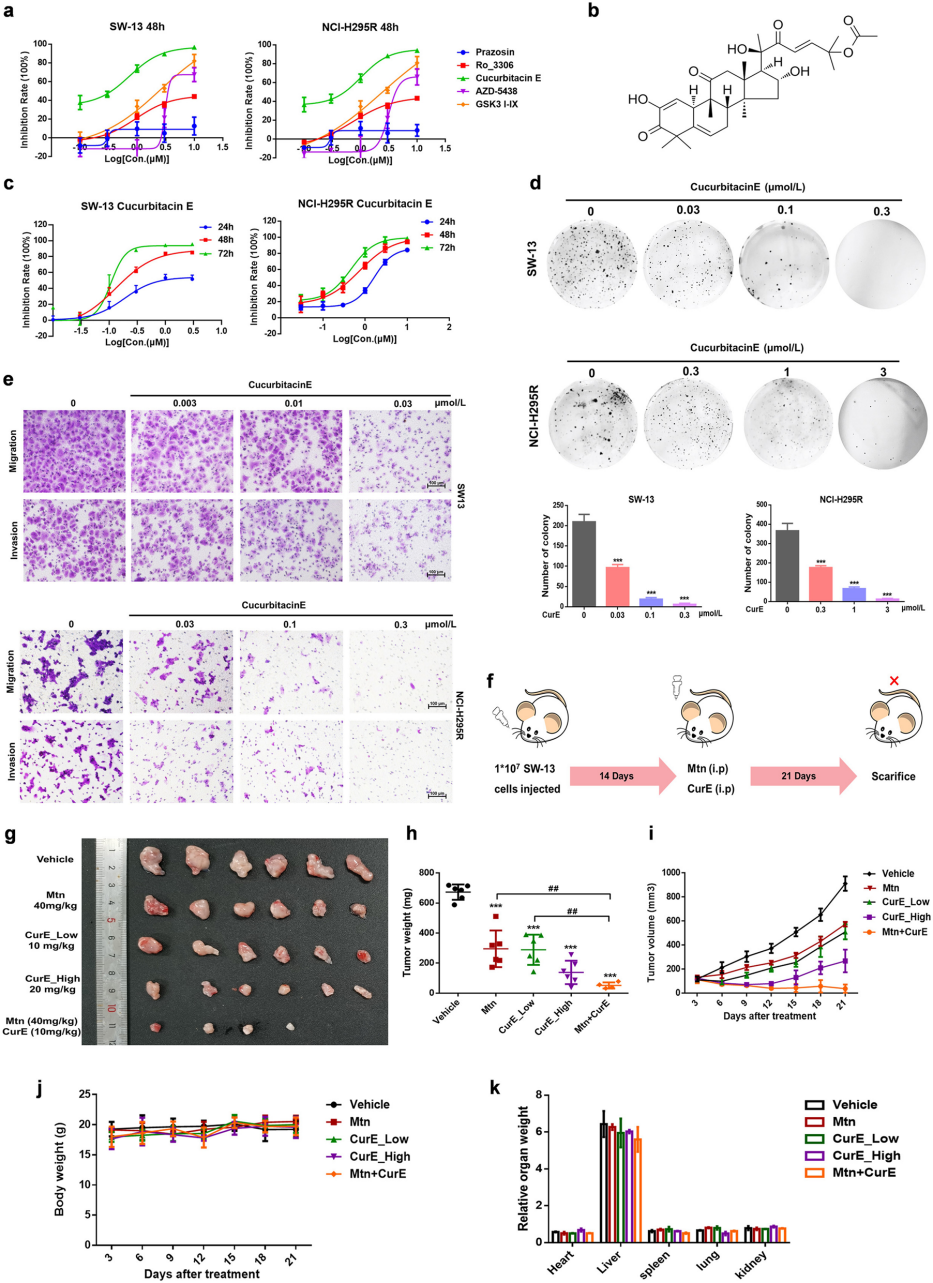
CDK1 inhibitor CurE inhibited proliferation of ACC cells in vitro and in vivo. **a** IC_50_ values (CCK-8 assay) of CDK1 inhibitors indicated that CurE had the best dose-dependent inhibitory activity in SW-13 and NCI-H295R cells. **b** Chemical structure of CurE. **c** IC_50_ values of CurE on SW-13 cells at 24, 48 and 72 h were 0.19, 0.13 and 0.11 μmol/L, respectively. IC_50_ values of CurE on NCI-H295R cells at 24, 48 and 72 h were 1.632, 0.732 and 0.464 μmol/L respectively. **d** CurE dose-dependently suppressed the invasion and migration of SW-13 and NCI-H295R cells. **e** CurE dose-dependently suppressed soft agar colony formation of SW-13 and NCI-H295R cells. **f** Diagram of xenograft experiment in nude mice for assessing antitumor effects of CurE. **g** Images of xenograft tumors of each group. Animal experiments were grouped as follows: vehicle, Mtn (40 mg/kg), CurE (10 mg/kg), CurE (20 mg/kg) and co-administration of Mtn (40 mg/kg) and CurE (10 mg/kg). **h–i** CurE dose-dependently reduced tumor weight **h** and tumor volume **i**, and its inhibitory effect was greater than that of the positive drug, Mtn; tumors in the drug combination group almost disappeared. **j** Body weight remained stable during drug administration in each group. **k** Relative organ weights showed no significant differences between groups. Data was shown as mean ± SD. ^##^*P* < 0.01 vs. Mtn group, ***P < 0.001, **P < 0.01, *P < 0.05 vs. vehicle group

To further evaluate the potential of CDK1 as a therapeutic target for ACC, we screened several CDK1 inhibitors for antitumor activity on ACC cell lines. Results revealed that CurE had the best inhibitory activity with good dose-dependency on SW-13 and NCI-H295R cells (Fig. 3a). Subsequent experiments verified that CurE (Fig. 3b) could time- and dose-dependently suppress the growth of NCI-H295R and SW-13 cells at nanomolar concentrations (Fig. 3c). CurE also inhibited colony formation of SW-13 and NCI-H295R cells (Fig. 3d) in the soft agar assay and suppressed their invasion and migration (Fig. 3e). CurE was tested in a nude mouse xenograft model of ACC (Fig. 3f) and it significantly and dose-dependently suppressed tumor growth in comparison with the vehicle group. The tumor weight and volume were reduced after treatment of CurE, and the therapeutic effect of 20 mg/kg of CurE (ip) was significantly better than that of the positive control drug, mitotane, at 40 mg/kg (ip). More importantly, the tumors were almost gone after 21 days of co-administration of 10 mg/kg CurE and 40 mg/kg mitotane (Fig. 3g–i). The body weight (Fig. 3j), morphological characteristics of organs including liver, heart, kidney, spleen, and lung remained stable (Fig. 3k) under the administration of CurE. No noticeable toxicity was identified through physiological blood tests (Additional file 3: Fig. S3). From the above results, we conclude that CurE can suppress the proliferation of ACC cells without observable toxic effects.

### Differential gene expression and enrichment analysis of SW-13 cells after treatment with CurE

To investigate the molecular mechanism of CurE’s antitumor effect against ACC, RNA-seq was carried out. We found 115 downregulated genes and 274 upregulated genes in SW-13 cells treated with CurE (Additional file 4: Fig. S4a). GO enrichment of the DEGs suggested that CurE principally regulated the apoptotic signaling pathway, the G2/M phase transition in the cell cycle, epithelial cell–cell adhesion, cell proliferation, and angiogenesis pathways (Additional file 4: Fig. S4b). The KEGG pathway enrichment indicated that CurE could regulate the cell cycle, the synthesis and metabolism of amino acids, cell adhesion molecules, P53 and PPAR signaling pathway, DNA replication, necroptosis and cellular senescence (Additional file 4: Fig. S4c). Reactome enrichment suggested that CurE participated in the S phase, G2/M checkpoints, MyD88-related pathway, CDK-mediated phosphorylation, antigen processing, proteasome degradation, apoptosis and programmed cell death (PCD) (Additional file 4: Fig. S4d). Overall, these results suggested that CurE could inhibit ACC cells by regulating the cell cycle, metastasis, and PCD.

#### CDK1 could regulate the G2/M phase transition in ACC cells

**Fig. 4:**
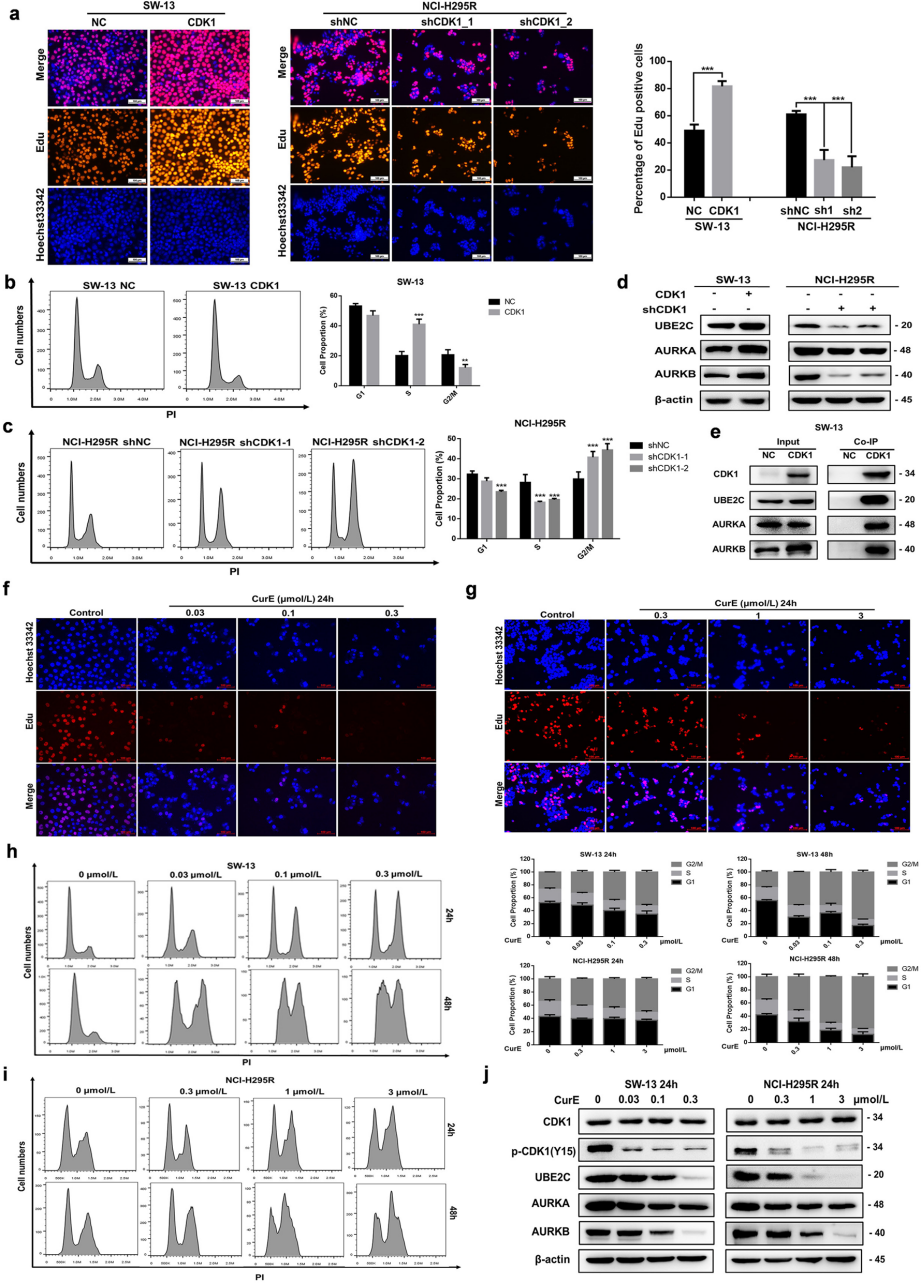
CDK1 regulated G2/M phase transition of ACC cells through interaction with UBE2C and AURKA/B. **a** Overexpression of CDK1 promoted DNA synthesis of SW-13 cells, while CDK1 knockdown suppressed DNA synthesis in NCI-H295R cells. **b** Overexpression of CDK1 reduced the percentage of G2/M phase cells and increased S phase in SW-13 cells. **c** Knockdown of CDK1 expression blocked the G2/M transition of NCI-H295R cells. **d** CDK1 altered the expression of UBE2C and AURKA/B. **e** CDK1 interacted with UBE2C and AURKA/B. **f** CurE suppressed DNA synthesis of SW-13 cells. **g** CurE suppressed DNA synthesis of NCI-H295R cells. **h** CurE time- and dose-dependently suppressed the G2/M phase in SW-13 cells. **i** CurE time- and dose-dependently suppressed the G2/M phase of NCI-H295R cells. **j** CurE blocked phosphorylation of CDK1 and reduced UBE2C and AURKA/B expression. Experiments were performed in triplicate, and data was presented as mean ± SD. **P < 0.01, ***P < 0.001 vs. control group

The Edu assay revealed that CDK1 could enhance DNA synthesis activity in ACC cells (Fig. 4a). Overexpression of CDK1 reduced the percentage of G2/M phase cells and increased the number of SW-13 cells in S phase (Fig. 4b). Knockdown of CDK1 arrested the NCI-H295R cells in G2/M phase (Fig. 4c). Western blotting and co-IP experiments showed that CDK1 could interact with the mitosis-associated proteins, UBE2C and AURKA/B, and regulate their expression (Fig. 4d–e). The protein stabilities of UBE2C and AURKA/B were significantly elevated in CDK1-overexpressing cells (Additional file 5: Fig. S5a–d). The effects of CurE on cell cycle in ACC cells were identified by flow cytometry and CurE was shown to reduce DNA synthesis in SW-13 (Fig. 4f) and NCI-H295R cells (Fig. 4g) in a dose-dependent way. CurE could time- and dose-dependently block the G2/M phase of the cell cycle of SW-13 (Fig. 4h) and NCI-H295R (Fig. 4i). In addition, CurE dose-dependently inhibited the expression of UBE2Cand AURKA/B. Thus, we concluded that CDK1 regulated the G2/M phase in ACC cells by regulating UBE2C and AURKA/B.

### CDK1 inhibitor CurE can induce PANoptosis in ACC cells

**Fig. 5:**
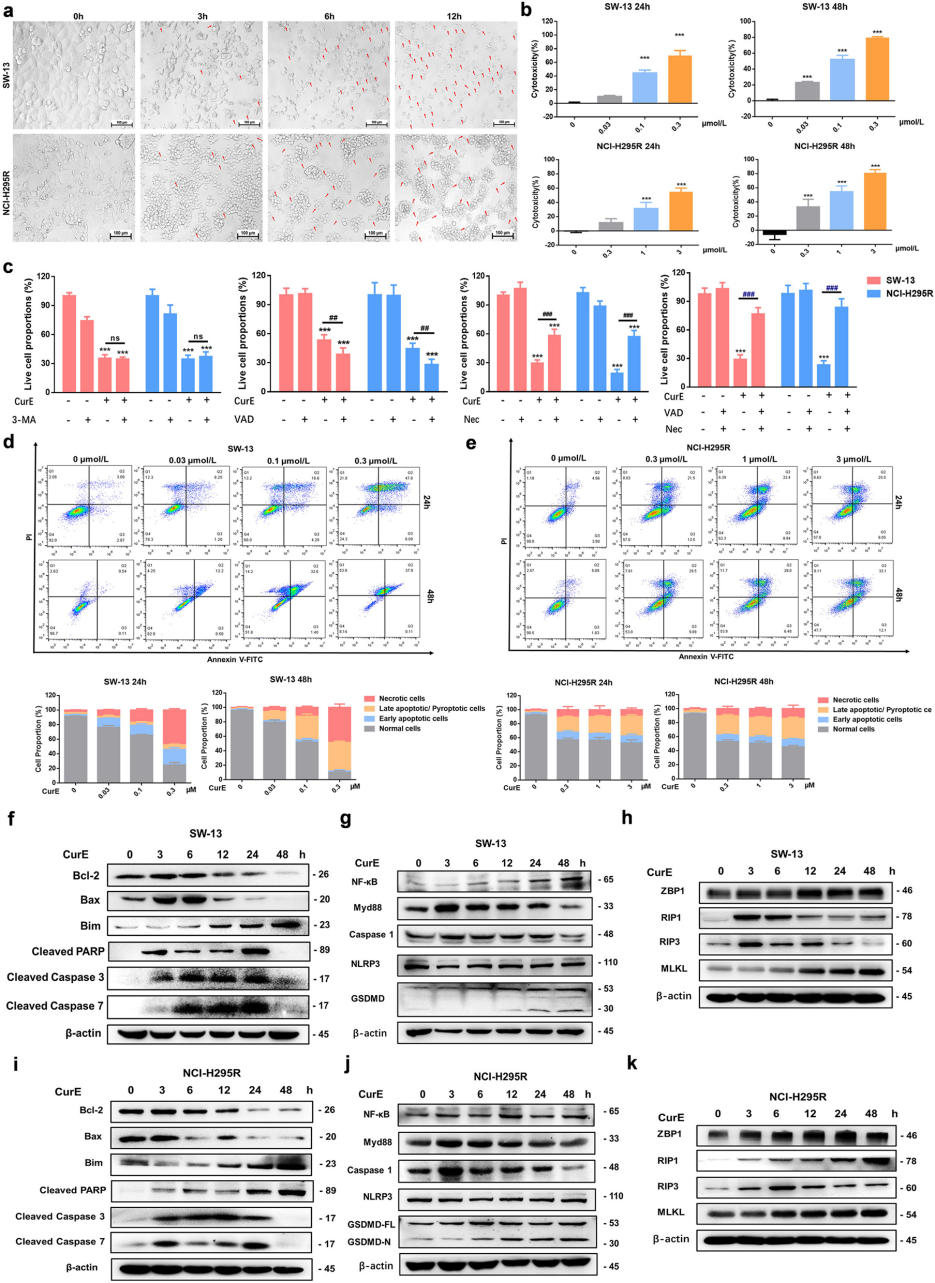
CDK1 inhibitor CurE induced apoptosis, pyroptosis, and necroptosis (PANoptosis) in ACC cells. **a** Effect of CurE on morphology of NCI-H295R and SW-13 cells at 3, 6 and 12 h. Spherical membranal protrusions were observed in NCI-H295R and SW-13 cells. **b** CurE caused the release of LDH from SW-13 and NCI-H295R cells. **c** The cytotoxicity of CurE could be partially rescued by combined treatment with Z-VAD-FMK (VAD) and necrosulfonamide (Nec), while it could not be restored by using them alone or using autophagy inhibitor 3-MA. **d, e** Annexin V-PI double staining showed that CurE increased the percentage of necrotic and apoptotic/pyroptotic SW-13 cells **d** and NCI-H295R **e** cells. **f, i** CurE time-dependently increased the expression of apoptosis marker protein, cleaved caspase 3/7, and altered the expression of other apoptosis-related proteins in SW-13 (**f**) and NCI-H295R (**i**) cells. **g, j** CurE time-dependently increased the expression of pyroptosis marker protein, GSDMD, and altered the expression of other pyroptosis-related proteins in SW-13 (**g**) and NCI-H295R (**j**) cells. **h, k** CurE time-dependently increased the expression of necroptosis marker protein, MLKL, and altered the expression of other necroptosis-related proteins in SW-13 (**h**) and NCI-H295R (**k**) cells. Experiments were performed in triplicate, and data was presented as mean ± SD. ***P < 0.001 vs. control group

The effect of CurE on morphology was observed in NCI-H295R and SW-13 cells at 3, 6 and 12 h. Spherical membrane protrusions were observed in NCI-H295R and SW-13 cells (Fig. 5a). CurE time- and dose-dependently promoted the release of LDH in SW-13 and NCI-H295R cells (Fig. 5b), corresponding to increased damage to the plasma membrane allowing leakage. The cytotoxicity of CurE could be partially rescued by co-administration of the apoptosis/pyroptosis inhibitor, VAD, and the necroptosis inhibitor, Nec, but it could not be reversed by using them alone or using the autophagy inhibitor, 3-MA (Fig. 5c). PI-annexin V staining was used to determine the type of cell death. The early phase of apoptosis was represented by single-positive staining for annexin V, while the late phase of apoptosis or pyroptosis showed double-positive staining [21]. Annexin V-PI double staining suggested that CurE time- and dose-dependently increased the percentage of necrotic and pyroptotic/apoptotic cells of SW-13 (Fig. 5d) and NCI-H295R (Fig. 5e).

Western blotting results indicated that the anti-apoptotic protein, Bcl-2, was remarkably reduced after treatment with CurE, while the pro-apoptotic proteins, Bax and Bim, were elevated in comparison with the control group. CurE increased the expression level of cleaved caspase 3 (CASP3), cleaved PARP and cleaved caspase 7 (CASP7) in SW-13 (Fig. 5f) and NCI-H295R (Fig. 5i) cells in a time-dependent manner. The characteristics of pyroptosis include gasdermin family-regulated pore generation in the cytomembrane, cell turgescence, and cytomembrane disruption, together with leakage of the proinflammatory cytokines [22]. Caspase 1 could cleave gasdermin D (GSDMD-FL) to produce an N-terminal cleavage product (GSDMD-N) under activation, which could induce pyroptosis by generating cytomembrane pores and leaking inflammatory cytokines [21]. Nuclear factor-κB (NF-κB) chiefly facilitates the production of proinflammatory effectors, like NLRP3, Myd88 and caspase 1 [23]. The results also indicated that CurE time-dependently elevated the expression of NF-κB and caspase 1 in SW-13 (Fig. 5g) and NCI-H295R (Fig. 5j) cells and significantly upregulated the expression of GSDMD-N. After treatment with CurE, the expression of Myd88 was found to be significantly increased, whereas no significant changes were seen in NLRP3.

Necroptosis is a caspase-independent necrotic cell death, which is mediated by strictly regulated cellular signaling pathways. It is chiefly regulated via ﻿receptor-interacting protein 3 (RIP3), RIP1, and mixed lineage kinase domain-like (MLKL) [24]. ZBP1 can induce PANoptosis of cells when it senses the Z-RNA produced during infection with influenza virus [25]. Results of Western blotting suggested that the expression levels of RIP1, RIP3 and MLKL were remarkably elevated after administration of CurE, and that the ZBP1 expression was significantly elevated in comparison with the control group (Fig. 5h, k). Based on the above results, CurE could induce PANoptosis of ACC cells in a caspase-independent way.

### CDK1 regulated the PANoptosis of ACC cells through binding with the PANoptosome in a ZBP1-dependent way

**Fig. 6:**
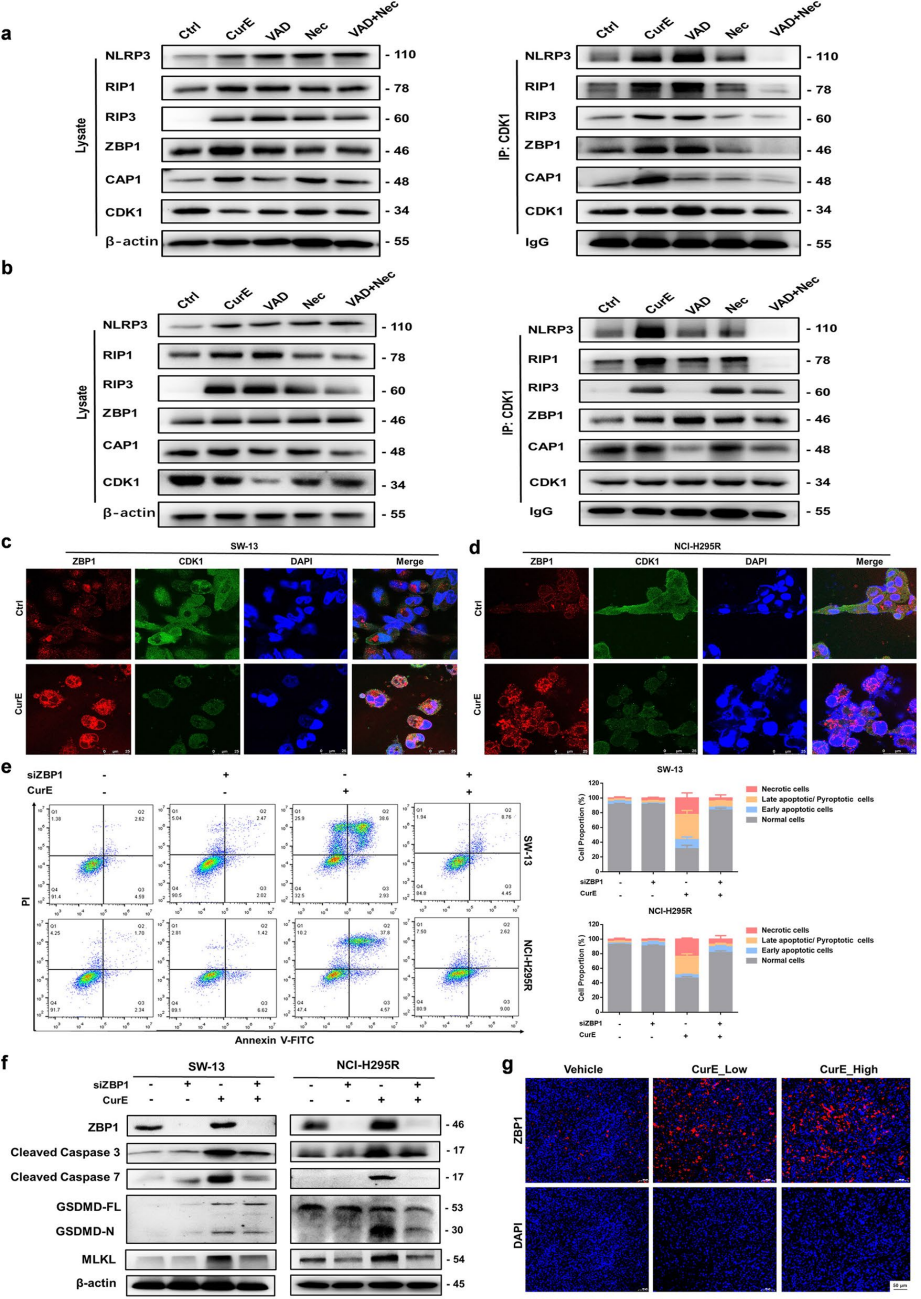
CDK1 regulated the PANoptosis of ACC cells through binding to the PANoptosome in a ZBP1-dependent way. **a** Co-IP data showed that CDK1 could bind to the PANoptosome in SW-13 cells, and **b** in NCI-H295R cells. **c** Immunofluorescence showed that CDK1 co-localized with ZBP1 in SW-13 cells, and **d** in NCI-H295R cells. **e** Silencing ZBP1 could rescue cell death triggered by CurE in SW-13 and NCI-H295R cells. **f** Silencing ZBP1 inhibited the marker proteins of apoptosis, pyroptosis, and necroptosis. **g** Immunofluorescence showed that ZBP1 was upregulated by administration of CurE in vivo

To further explore the mechanism of CDK1 in regulating PANoptosis, we evaluate the relationship between CDK1 and the PANoptosome. Co-IP experiments showed that CurE could induce the formation of PANoptosomes and that CDK1 could bind to the PANoptosome in SW-13 cells (Fig. 6a) and NCI-H295R cells (Fig. 6b). Immunofluorescence experiments showed that CDK1 co-localized with ZBP1 in SW-13 (Fig. 6c) and NCI-H295R cells (Fig. 6d). Silencing ZBP1 could rescue the cell death triggered by CurE in SW-13 and NCI-H295R cells (Fig. 6e). And when ZBP1 was knockdown, it could inhibit the marker proteins of apoptosis, pyroptosis, and necroptosis which were triggered by CurE (Fig. 6f). In addition, when caspase 3 was knocked down, the percentage of necrotic cells was raised after treatment with CurE, whereas the overall cell survival rate didn’t significantly change in NCI-H295R and SW-13 cells. Similarly, when GSDMD was knocked down, the proportion of necrotic cells was increased, but the overall cell survival didn’t change significantly (Additional file 6: Fig. S6a–h). The above results indicated that when the pathways of apoptosis and pyroptosis were inhibited, CurE was able to suppress the proliferation of ACC cells via triggering cell necroptosis instead. These results also suggested that CurE inhibited the proliferation of ACC cells through triggering PANoptosis. Furthermore, immunofluorescence experiments also showed that ZBP1 was upregulated with administration of CurE in vivo (Fig. 6g). In addition, the experimental results showed that knockdown of CDK1 could significantly reverse the inhibitory effect of CurE on ACC cell lines (Additional file 7: Fig. S7a–f), which indicated that the above effects of CurE were due to the CDK1 inhibition. In conclusion, CDK1 regulated the PANoptosis of ACC cells through binding with the PANoptosome in a ZBP1-dependent way.

## Discussion

ACC is an aggressive and extremely rare endocrine neoplasm with poor prognosis [26]. So far, mitotane is the only approved therapeutic drug for ACC since 1970 despite its suboptimal efficacy [27]. No new drug has been developed for ACC since 1970. Thus, it is critically necessary to identify novel therapeutic targets and drugs for ACC. Our experimental results showed that CDK1 could be an essential prognostic and therapeutic target of ACC via regulation of EMT, G2/M phase and PANoptosis. Thus, CurE with its good record of safety and efficacy is a likely candidate for ACC therapy, which will provide much-needed help for patients with ACC.

There are few studies on ACC and its therapeutic targets because ACC is such a rare endocrine malignant disease. It was reported that TOP2A was abnormally elevated in ACC and regulated cell growth and metastasis of ACC cells [28]. CDK4 was highly expressed in ACC and several CDK4 inhibitors could decrease the growth rate of ACC cell lines in vitro [29]. However, none of these studies have made further progress and the treatment situation of ACC is still grim. As CDK1 was abnormally elevated in the majority of tumors, we explored its expression pattern in ACC and found that CDK1 expression was significantly elevated. Various bioinformatics analysis studies also pointed out that CDK1 was upregulated in ACC with prognostic significance [30–32]. One study suggested that the overexpressed centromere protein F (CENPF)/CDK1 signaling pathway enhanced the progression of ACC via regulating the G2/M-phase cell cycle [33]. Therefore, we proposed that CDK1 might become a promising target for ACC treatment.

Our results demonstrated that CDK1 was significantly overexpressed in ACC and that the expression level of CDK1 was correlated with the pathological stage and nodal metastasis status of ACC with prognostic significance. The upregulation of CDK1 facilitated the growth and metastasis of ACC cells, whereas CDK1 knockdown had the opposite effect. RNA sequence and CO-IP experiments showed CDK1 interacting with mitosis-associated protein UBE2C [34], AURKA [35] and AURKB, and with Slug and Twist, which are essential proteins in metastatic signaling. In view of these findings, we propose that CDK1 plays a vital part in the growth and metastasis of ACC and therefore should be a promising target for ACC therapy.

Almost five decades ago, mitotane was approved as the only first-line therapeutic drug of ACC. However, the prognosis of ACC patients remains unsatisfactory and new effective drugs for ACC are still in urgent need of development [36]. Targeting CDK1 has been a promising antitumor approach for drug development. Numerous phase II/III and phase I clinical trials have been conducted to test the effect and assess the safety of CDK1 inhibitors. The PLK1 inhibitor, rigosertib, entered the clinical research stage at phase II/III for the treatment of pancreatic cancer and glioma [6, 7]. Through screening CDK1 inhibitors in ACC cell lines, we established that CurE had the best inhibitory effect with clear time- and dose-dependency. Other studies showed that CurE was able to chemo-sensitize colon tumor cells by regulating TFAP4/Wnt/beta-catenin signaling [37] and inhibit the proliferation of pancreatic adenocarcinoma cells by modulating STAT3 signaling [38]. CurE also suppressed the brain metastasis of lung carcinoma cells in vivo by inhibiting the Yes-related signaling pathway [39]. Our results first found that CurE suppressed cell growth and metastasis of ACC cells in vitro at nanomolar concentrations, and that it could induce PANoptosis in ACC cells in a ZBP1-dependent and caspase-independent way. In addition, administration of CurE inhibited ACC tumor proliferation in vivo without noticeable toxic effects in a nude mouse xenograft model and showed better therapeutic effect than mitotane, which is the only drug currently approved for ACC treatment. More importantly, the combined treatment of CurE with mitotane nearly eliminated the ACC xenograft tumors. These results support the idea that CurE is a promising drug candidate for the treatment of ACC that could be used in combination with mitotane.

Programmed cell death (PCD), which is mediated via an evolutionarily conserved signaling pathway, is one of the main targets in the antitumor drug search [13]. A novel identified pathway for PCD called PANoptosis is mediated through a PANoptosome, which is a newly recognized multimeric cytoplasmic protein complex [14]. The PANoptosome incorporates three crucial effectors of PCD in parallel: pyroptosis, apoptosis, and necroptosis [15]. Our results show that CDK1 can interact with the PANoptosome and that the CDK1 inhibitor induces PANoptosis of ACC cells in a ZBP1-dependent way. PANoptosis is a new mechanism for antitumor drug activity, and has not yet been reported by other antitumor drug-development researchers. PANoptosis can produce cell death in several different ways, so a tumor cell might not be able to develop resistance to CurE that easily. Also, we found that the therapeutic effect of a combination of CurE and mitotane was significantly greater than either alone, which means that more patients could be benefitted in clinical practice. ACC is a very rare malignant cancer that has received little attention from researchers and CurE may be the solution to meet the crucial needs of patients with ACC throughout the world.

## Conclusions

**Fig. 7:**
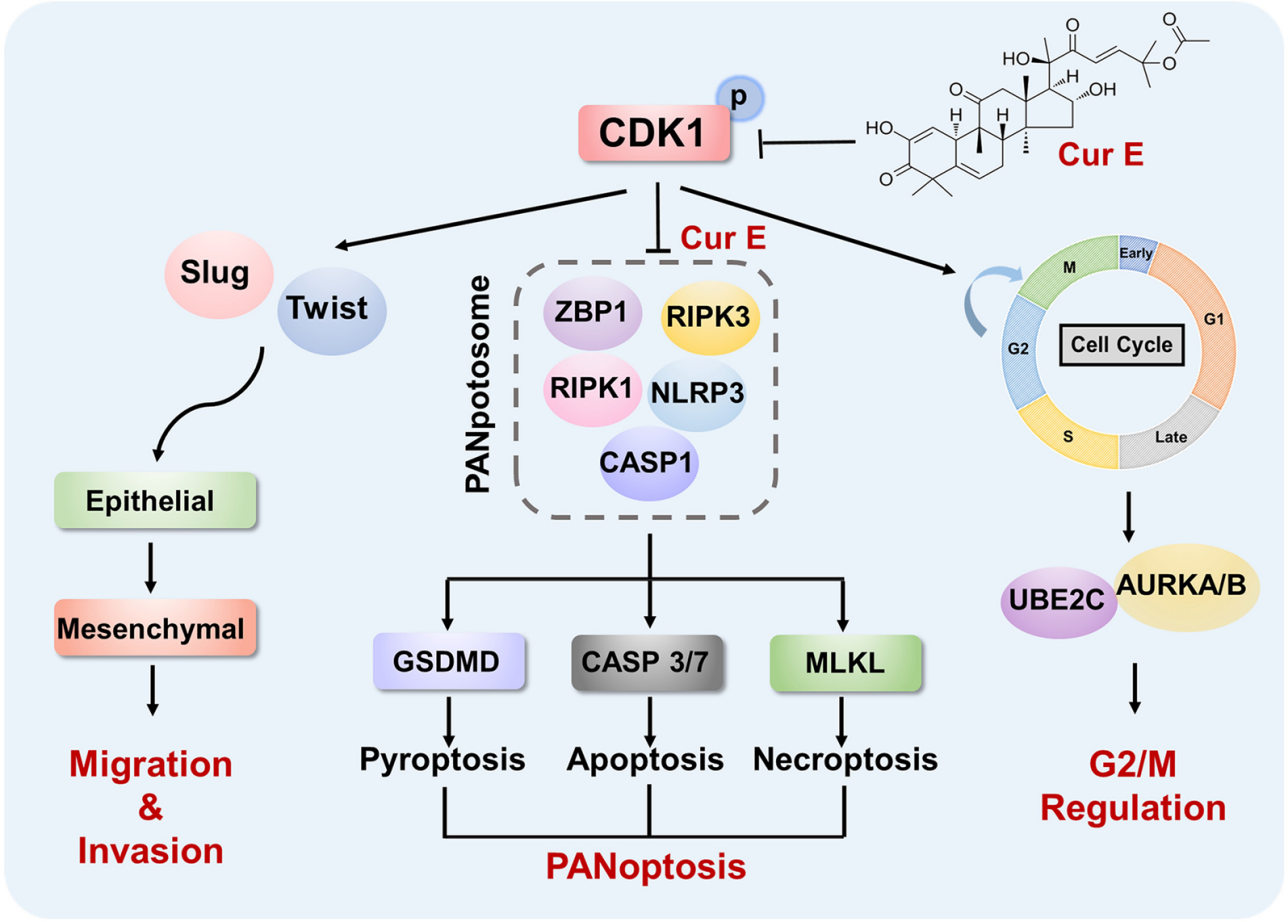
The role of CDK1 in ACC. CDK1 serves as a therapeutic target of adrenocortical carcinoma as a regulator of the epithelial-mesenchymal transition, G2/M phase, and PANoptosis. CurE is a probable candidate for the treatment of ACC by inhibiting CDK1

In conclusion, CDK1 facilitated the EMT of ACC cells via Slug and Twist and regulated G2/M phase of ACC cells through regulating UBE2C and AURKA/B. CDK1 also mediated PANoptosis of ACC cells through binding to the PANoptosome in a ZBP1-dependent way. The CDK1 inhibitor CurE was found to significantly inhibit ACC cell proliferation and metastasis at low concentration both in vitro and in vivo with good safety and efficacy. Most importantly, the combined treatment of CurE and mitotane virtually eliminated ACC tumors in nude mice. We conclude that CDK1 may serve as an essential prognostic and therapeutic target of ACC (Fig. 7). CurE is expected to be approved as the next potential drug for the treatment of ACC, especially in combination with mitotane.

## Supplementary Information

**Additional file 1: Figure S1.** CDK1 is a potential target and prognostic biomarker of ACC. a CDK1 was abnormally upregulated in multiple types of tumors as analyzed on the Broad institute website. b CDK1 was abnormally upregulated in multiple types of tumors as analyzed on the Oncomine website. c CDK1 was highly expressed in ACC as analyzed via TCGA database. d CDK1 expression was increased with the clinical grade of ACC. e CDK1 expression increased the higher the nodal metastasis status of ACC, compared to non-metastatic ACC. f–h The Kaplan-Meier survival analysis suggested that CDK1 expression was strongly related to the OS, DSS and PFI survival probability of ACC patients. i–k The ROC curve of CDK1 expression and OS, DSS and PFI survival suggested that CDK1 could be useful as a prognostic indicator of ACC.

**Additional file 2: Figure S2.** Differential gene expression and enrichment analysis of overexpressed CDK1 in SW-13 cells. a CDK1 expression was higher in NCI-H295R than in SW-13 cells. b Volcano plot of DEGs between SW-13_CDK1 and SW-13_NC cells. 104 genes were downregulated and 66 genes were overexpressed in the SW-13_CDK1 group, compared to the SW-13_NC group. c Gene ontology enrichment of DEGs suggested that CDK1 participated in cell-cell adhesion, muscle structure development, angiogenesis and cell proliferation. d KEGG pathway enrichment of DEGs suggested that CDK1 regulated the cell adhesion molecules, epithelial cell signaling, and cell cycle pathways. e Reactome pathway enrichment of DEGs indicated that CDK1 could play an essential part in GPCR signaling.

**Additional file 3: Figure S3.** Analysis of blood physiology parameters of control, vehicle- and CurE-treated mice. The following blood physiology functions were assayed: WBC: white blood cell count; RBC: red blood cell count; HGB: hemoglobin; MCV: mean corpuscular volume; RDW: red blood cell volume distribution width; PLT: platelet; LYM: lymphocyte count; MON: monocyte count; NEUT: neutrophil count. The data were shown as mean ± standard deviation. N = 6 for each group, **P < 0.01 vs. control group.

**Additional file 4: Figure S4.** Abnormal expressed gene and functional enrichment analysis of SW-13 cells after treatment of CurE. a Volcano map of differential genes of SW-13 cells after treatment of CurE. There were 115 downregulated genes and 274 upregulated genes identified in CurE treated SW-13 cells. b Gene ontology enrichment of DEGs revealed that CurE mainly affected apoptotic signaling pathway, epithelial cell-cell adhesion and angiogenesis pathways. c KEGG pathway enrichment suggested CurE regulated the cell cycle, the synthesis and metabolism of amino acids, cell adhesion molecules, P53 and PPAR signaling pathway, DNA replication, necroptosis and cellular senescence. d Reactome pathway enrichment suggested that CurE participated in the S phase, G2/M checkpoints, MyD88 related pathway, CDK- mediated phosphorylation, antigen processing, proteasome degradation, apoptosis and programmed cell death.

**Additional file 5: Figure S5.** Overexpression of CDK1 enhanced protein stabilities of UBE2C and AURKA/B. a Expressions of UBE2C and AURKA/B in SW-13_NC and SW-13_CDK1 cells after treatment with CHX (100 μmol/L) at 0, 1, 3, 9, 12 h. b Relative expression of UBE2C, AURKA c and AURKB d in SW-13_NC and SW-13_CDK1 cells after treatment with CHX. Statistical differences were assessed by two-way ANOVA. *P < 0.05, **P < 0.01, ***P < 0.001.

**Additional file 6: Figure S6.** Silencing the markers of apoptosis or pyroptosis could not rescue CurE-mediated cell death. a Transfection efficacy of knockdown caspase 3 expression in SW-13 cells. b Transfection efficacy of knockdown caspase 3 expression in NCI-H295R cells. c Transfection efficacy of knockdown GSDMD expression in SW-13 cells. b Transfection efficacy of knockdown GSDMD expression in NCI-H295R cells. e Silencing caspase 3 increased necrotic cells triggered by CurE in SW-13 cells. f Silencing caspase 3 increased necrotic cells triggered by CurE in NCI-H295R cells. g Silencing GSDMD increased necrotic cells triggered by CurE in SW-13 cells. h Silencing GSDMD increased necrotic cells triggered by CurE in NCI-H295R cells.

**Additional file 7: Figure S7.** Knockdown of CDK1 could significantly reverse the inhibitory effect of CurE on ACC cell lines. a Effect of CurE on morphology of CDK1-knockdown and control SW-13 and b NCI-H295R cells. c Effect of CurE on inhibition rate in CDK1-knockdown and control SW-13 and d NCI-H295R cells. e Effect of CurE on cell cycle in CDK1-knockdown and control SW-13 and f NCI-H295R cells. CDK1-knockdown and control cells were treated with 0.3 μmol/L CurE for 24 h in SW-13 cells and 1 μmol/L CurE for 24 h in NCI-H295R cells. Experiments were performed in triplicate, and data was presented as mean ± SD. ***P < 0.001 vs. control group.

**Additional file 8: Table S1.** Information of antibodies used in this research.

## Data Availability

The datasets generated during the current study are not publicly available due to privacy and confidentiality reasons but are available from the corresponding author on reasonable request.

## Abbreviations

ACC: Adrenocortical carcinoma
Mtn: Mitotane
TCGA: The cancer genome atlas
Co-IP: Co-immunoprecipitation
IF: Immunofluorescence
EMT: Epithelial-mesenchymal transition
CurE: Cucurbitacin E
PANoptosis: The apoptosis, pyroptosis, and necroptosis
FDA: Food and Drug Administration
CDK1: Cyclin-dependent kinase 1
MMPs: Metalloproteinases
PCD: Programmed cell death
ZBP1: Z-DNA binding protein 1
LDH: Lactate dehydrogenase
NEB: New England biolabs
GO: Gene ontology
IHC: Immunohistochemistry
IF: Immunofluorescence
SD: Standard deviation
OS: Overall survival
DSS: Disease specific survival
PFI: Progression free interval
CASP3: Cleaved caspase 3
DEGs: Differential expressed genes
CASP7: Cleaved caspase 7
GSDMD: Gasdermin D
NF-κB: Nuclear factor-κB
RIP3: ﻿Receptor-interacting protein 3
MLKL: Mixed lineage kinase domain-like
CHX: Cycloheximide
