# Supplementary figures and images for: CDK1 serves as a therapeutic target of adrenocortical carcinoma via regulating epithelial–mesenchymal transition, G2/M phase transition, and PANoptosis
Source: J Transl Med. 2022 Oct 2;20:444. doi: 10.1186/s12967-022-03641-y (PMC9528181; doi:10.1186/s12967-022-03641-y)

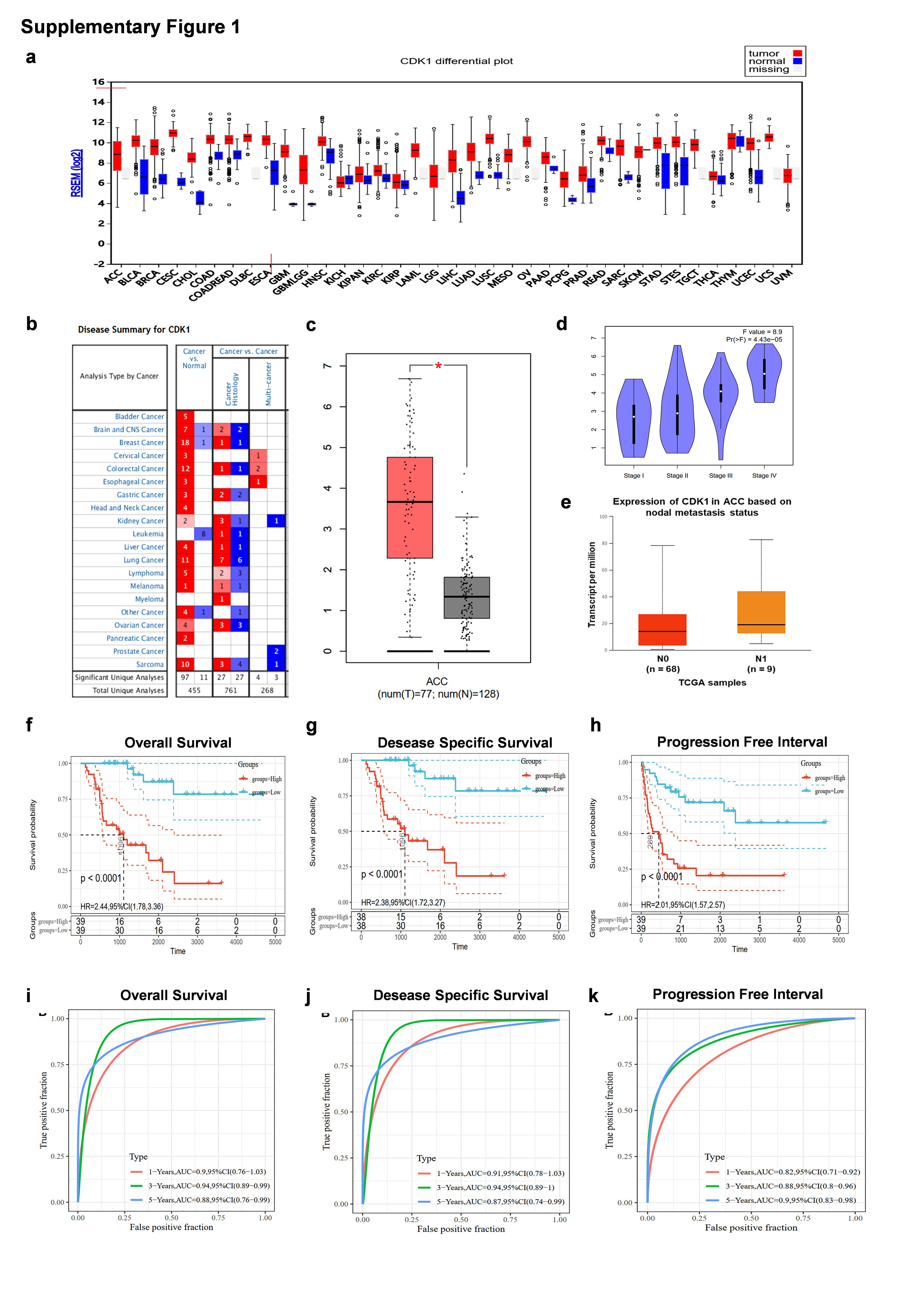

Supplement: Supplementary file 1 — Additional file 1: Figure S1. CDK1 is a potential target and prognostic biomarker of ACC. a CDK1 was abnormally upregulated in multiple types of tumors as analyzed on the Broad institute website. b CDK1 was abnormally upregulated in multiple types of tumors as analyzed on the Oncomine website. c CDK1 was highly expressed in ACC as analyzed via TCGA database. d CDK1 expression was increased with the clinical grade of ACC. e CDK1 expression increased the higher the nodal metastasis status of ACC, compared to non-metastatic ACC. f–h The Kaplan-Meier survival analysis suggested that CDK1 expression was strongly related to the OS, DSS and PFI survival probability of ACC patients. i–k The ROC curve of CDK1 expression and OS, DSS and PFI survival suggested that CDK1 could be useful as a prognostic indicator of ACC. [file 12967_2022_3641_MOESM1_ESM.jpg]

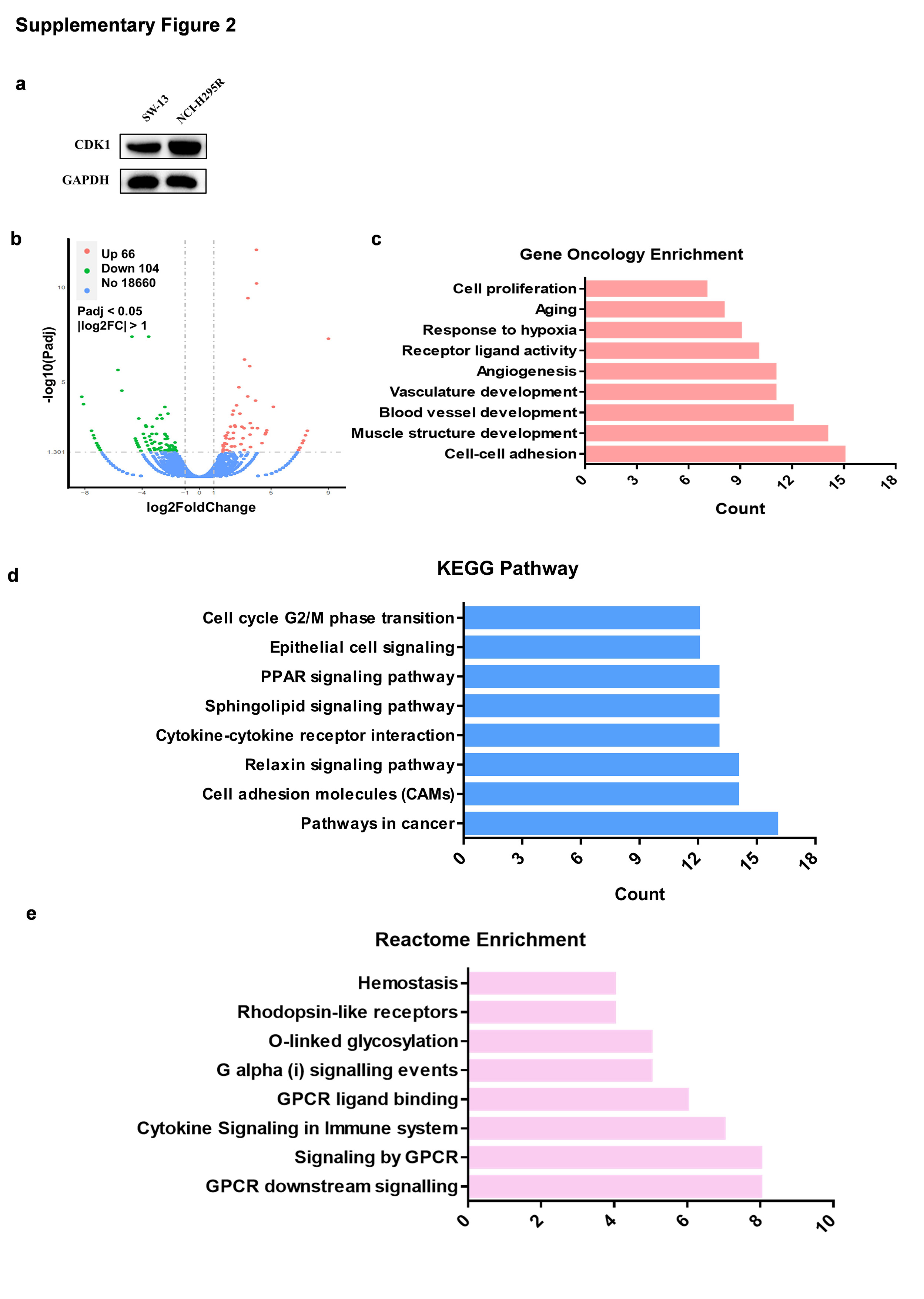

Supplement: Supplementary file 2 — Additional file 2: Figure S2. Differential gene expression and enrichment analysis of overexpressed CDK1 in SW-13 cells. a CDK1 expression was higher in NCI-H295R than in SW-13 cells. b Volcano plot of DEGs between SW-13_CDK1 and SW-13_NC cells. 104 genes were downregulated and 66 genes were overexpressed in the SW-13_CDK1 group, compared to the SW-13_NC group. c Gene ontology enrichment of DEGs suggested that CDK1 participated in cell-cell adhesion, muscle structure development, angiogenesis and cell proliferation. d KEGG pathway enrichment of DEGs suggested that CDK1 regulated the cell adhesion molecules, epithelial cell signaling, and cell cycle pathways. e Reactome pathway enrichment of DEGs indicated that CDK1 could play an essential part in GPCR signaling. [file 12967_2022_3641_MOESM2_ESM.jpg]

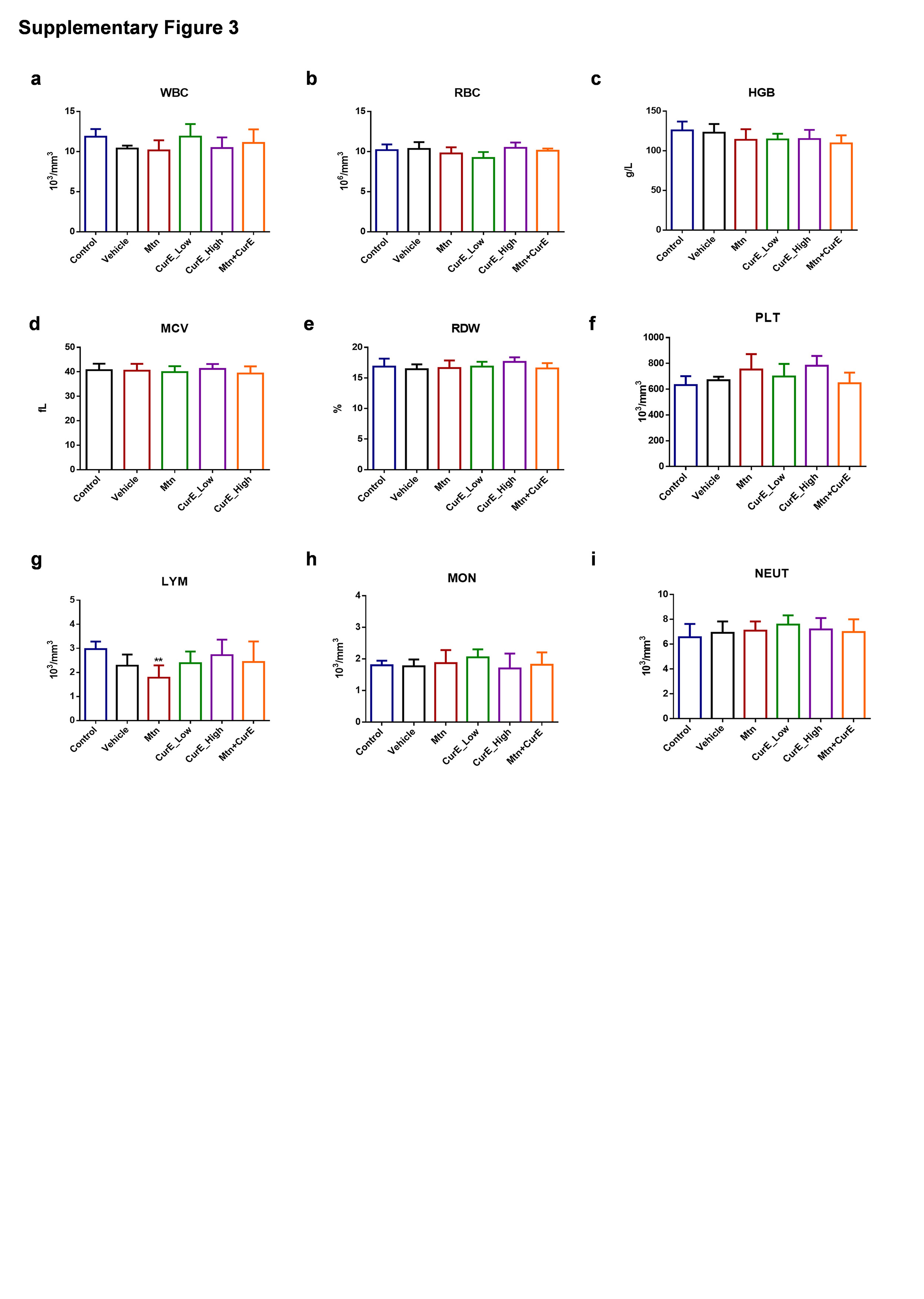

Supplement: Supplementary file 3 — Additional file 3: Figure S3. Analysis of blood physiology parameters of control, vehicle- and CurE-treated mice. The following blood physiology functions were assayed: WBC: white blood cell count; RBC: red blood cell count; HGB: hemoglobin; MCV: mean corpuscular volume; RDW: red blood cell volume distribution width; PLT: platelet; LYM: lymphocyte count; MON: monocyte count; NEUT: neutrophil count. The data were shown as mean ± standard deviation. N = 6 for each group, **P < 0.01 vs. control group. [file 12967_2022_3641_MOESM3_ESM.jpg]

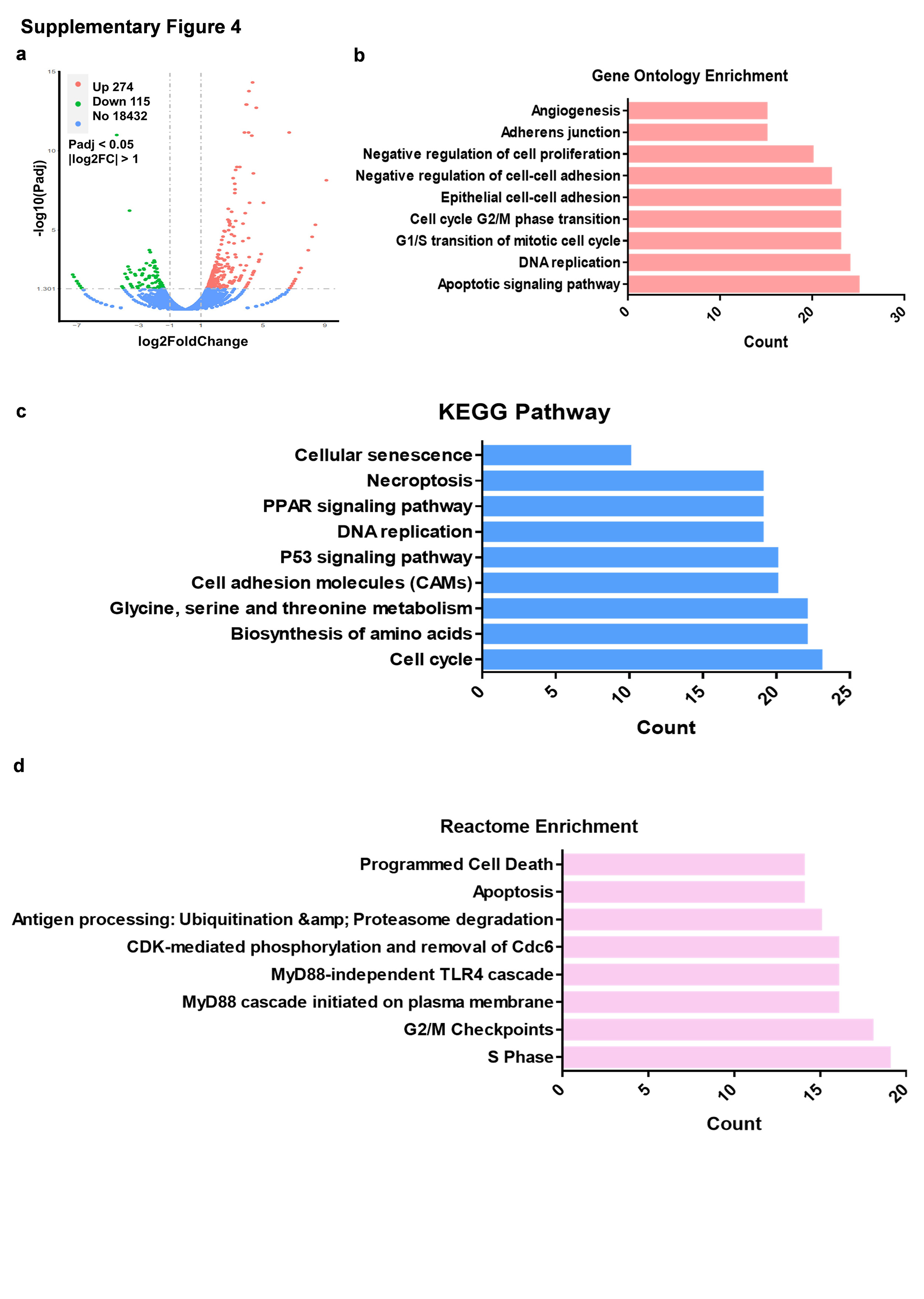

Supplement: Supplementary file 4 — Additional file 4: Figure S4. Abnormal expressed gene and functional enrichment analysis of SW-13 cells after treatment of CurE. a Volcano map of differential genes of SW-13 cells after treatment of CurE. There were 115 downregulated genes and 274 upregulated genes identified in CurE treated SW-13 cells. b Gene ontology enrichment of DEGs revealed that CurE mainly affected apoptotic signaling pathway, epithelial cell-cell adhesion and angiogenesis pathways. c KEGG pathway enrichment suggested CurE regulated the cell cycle, the synthesis and metabolism of amino acids, cell adhesion molecules, P53 and PPAR signaling pathway, DNA replication, necroptosis and cellular senescence. d Reactome pathway enrichment suggested that CurE participated in the S phase, G2/M checkpoints, MyD88 related pathway, CDK- mediated phosphorylation, antigen processing, proteasome degradation, apoptosis and programmed cell death. [file 12967_2022_3641_MOESM4_ESM.jpg]

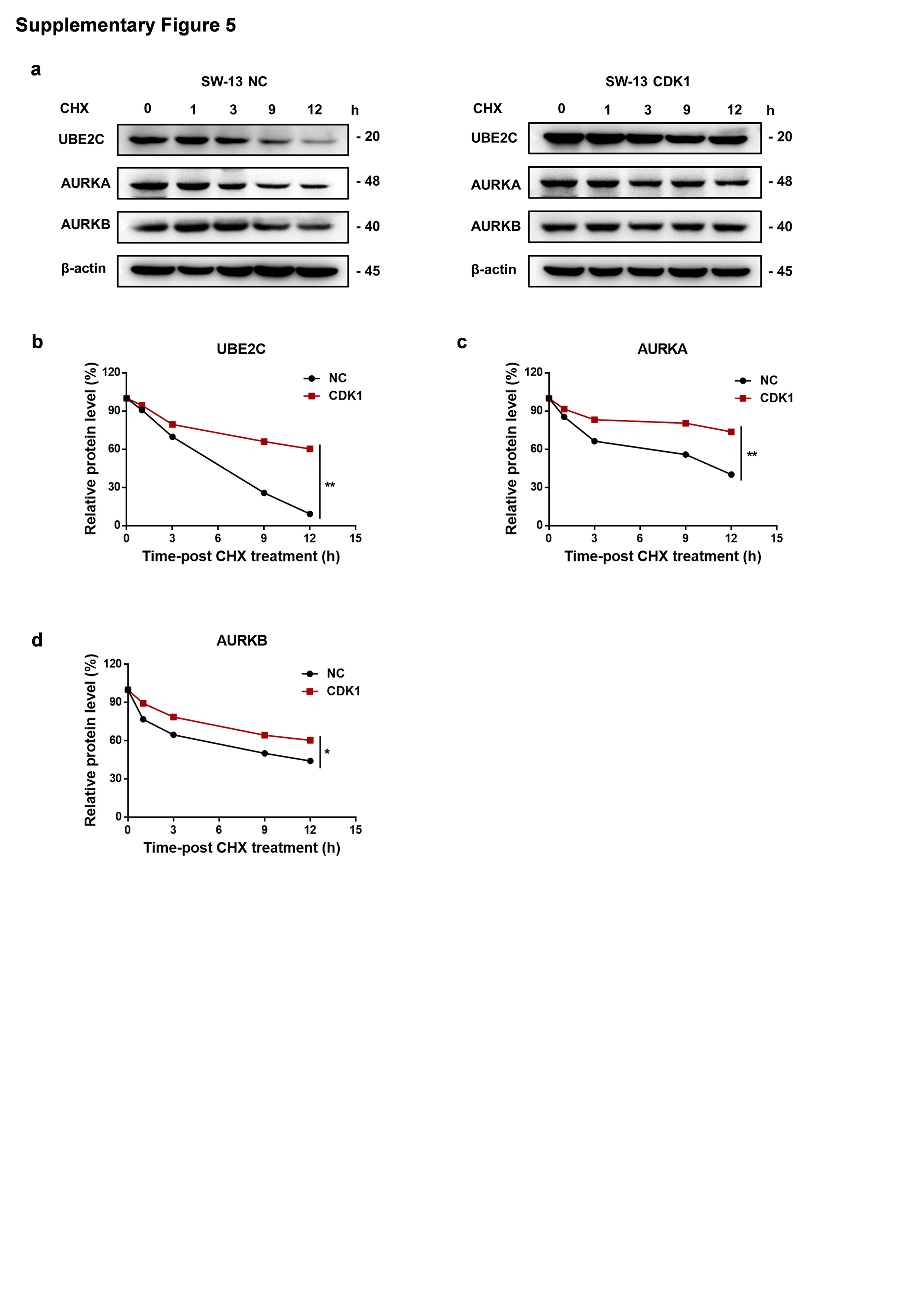

Supplement: Supplementary file 5 — Additional file 5: Figure S5. Overexpression of CDK1 enhanced protein stabilities of UBE2C and AURKA/B. a Expressions of UBE2C and AURKA/B in SW-13_NC and SW-13_CDK1 cells after treatment with CHX (100 μmol/L) at 0, 1, 3, 9, 12 h. b Relative expression of UBE2C, AURKA c and AURKB d in SW-13_NC and SW-13_CDK1 cells after treatment with CHX. Statistical differences were assessed by two-way ANOVA. *P < 0.05, **P < 0.01, ***P < 0.001. [file 12967_2022_3641_MOESM5_ESM.jpg]

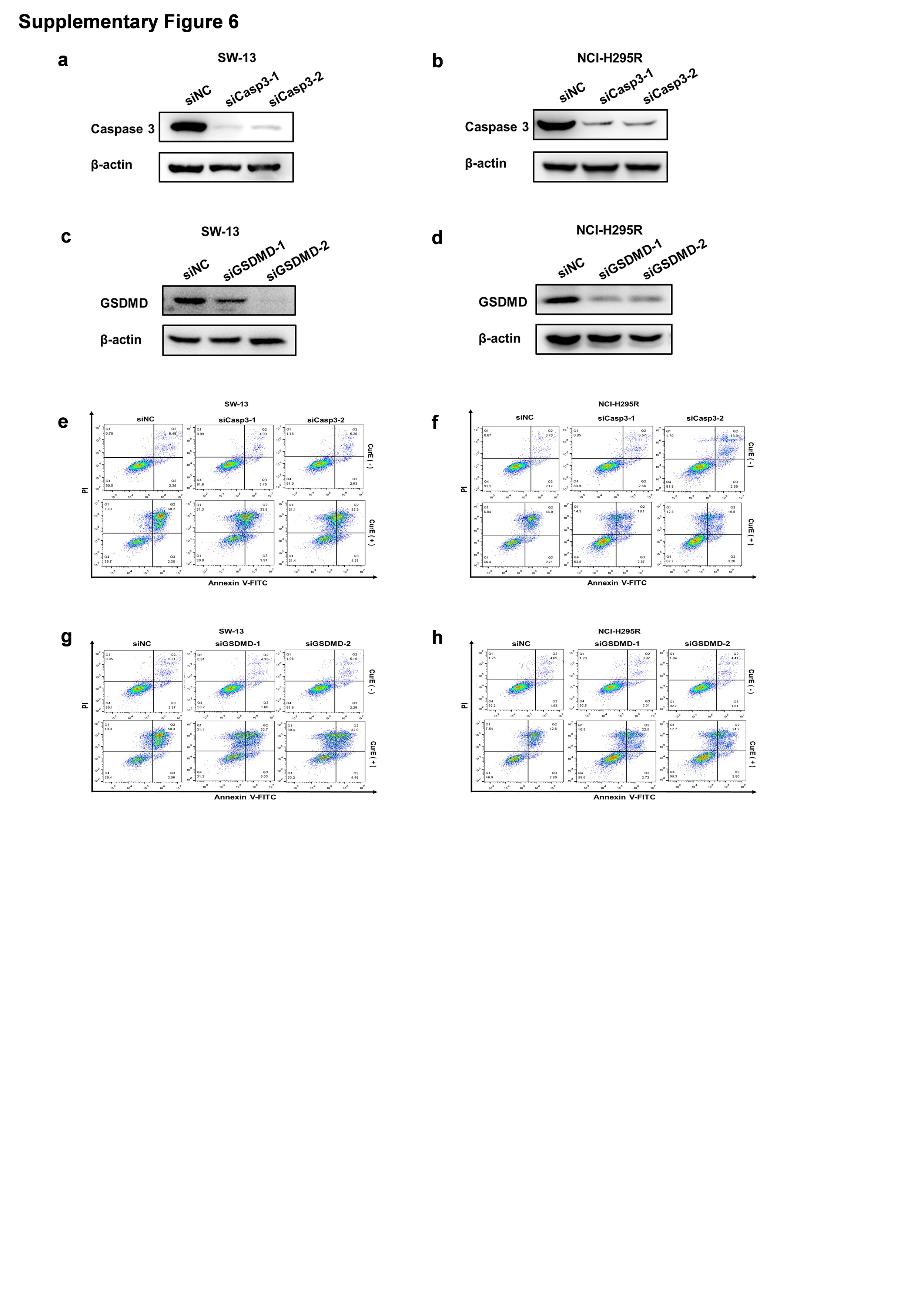

Supplement: Supplementary file 6 — Additional file 6: Figure S6. Silencing the markers of apoptosis or pyroptosis could not rescue CurE-mediated cell death. a Transfection efficacy of knockdown caspase 3 expression in SW-13 cells. b Transfection efficacy of knockdown caspase 3 expression in NCI-H295R cells. c Transfection efficacy of knockdown GSDMD expression in SW-13 cells. b Transfection efficacy of knockdown GSDMD expression in NCI-H295R cells. e Silencing caspase 3 increased necrotic cells triggered by CurE in SW-13 cells. f Silencing caspase 3 increased necrotic cells triggered by CurE in NCI-H295R cells. g Silencing GSDMD increased necrotic cells triggered by CurE in SW-13 cells. h Silencing GSDMD increased necrotic cells triggered by CurE in NCI-H295R cells. [file 12967_2022_3641_MOESM6_ESM.jpg]

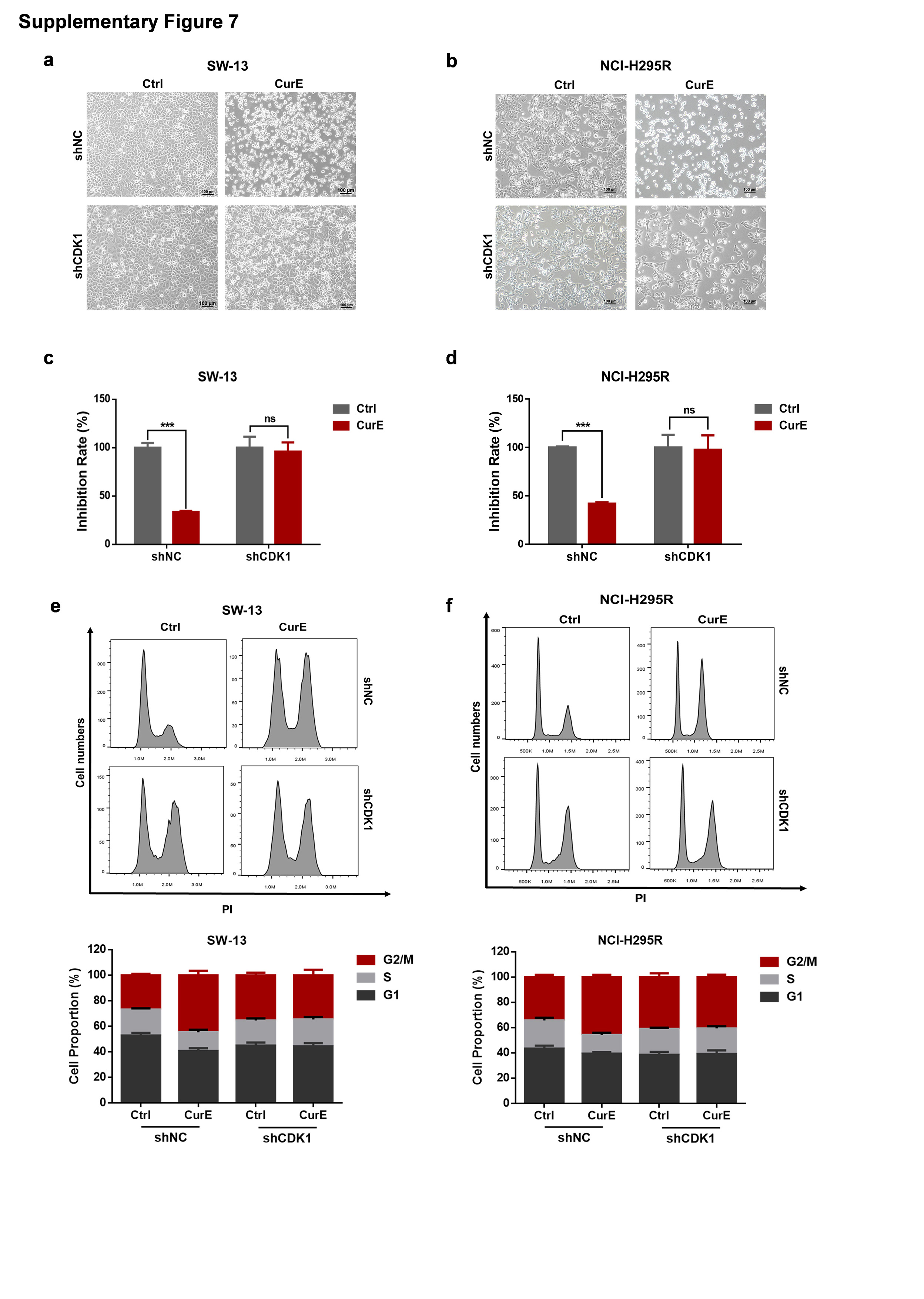

Supplement: Supplementary file 7 — Additional file 7: Figure S7. Knockdown of CDK1 could significantly reverse the inhibitory effect of CurE on ACC cell lines. a Effect of CurE on morphology of CDK1-knockdown and control SW-13 and b NCI-H295R cells. c Effect of CurE on inhibition rate in CDK1-knockdown and control SW-13 and d NCI-H295R cells. e Effect of CurE on cell cycle in CDK1-knockdown and control SW-13 and f NCI-H295R cells. CDK1-knockdown and control cells were treated with 0.3 μmol/L CurE for 24 h in SW-13 cells and 1 μmol/L CurE for 24 h in NCI-H295R cells. Experiments were performed in triplicate, and data was presented as mean ± SD. ***P < 0.001 vs. control group. [file 12967_2022_3641_MOESM7_ESM.jpg]
