# Supplementary material for: CDK1 serves as a therapeutic target of adrenocortical carcinoma via regulating epithelial–mesenchymal transition, G2/M phase transition, and PANoptosis
Source: J Transl Med. 2022 Oct 2;20:444. doi: 10.1186/s12967-022-03641-y (PMC9528181; doi:10.1186/s12967-022-03641-y)
Supplement: Supplementary file 8 — Additional file 8: Table S1. Information of antibodies used in this research. [file 12967_2022_3641_MOESM8_ESM.docx]

**Supplementary Table 1. Information of antibodies used in this research.**

| **Antibody** | **Catalogue** | **Dilution** | **Company** |
| --- | --- | --- | --- |
| β-actin | 66009-1-Ig | 1:5000 | Proteintech, Europe |
| CDK1 | 19532-1-AP | 1:1000 | Proteintech, Europe |
| CDC25C | 16485-1-AP | 1:1000 | Proteintech, Europe |
| MELK | 2274 | 1:1000 | Cell Signaling Technology, USA |
| UBE2C | 12134-2-AP | 1:1000 | Proteintech, Europe |
| AURKA | 14475 | 1:1000 | Cell Signaling Technology, USA |
| AURKB | 28711 | 1:1000 | Cell Signaling Technology, USA |
| Slug | 9585 | 1:1000 | Cell Signaling Technology, USA |
| Twist | 25465-1-AP | 1:1000 | Proteintech, Europe |
| Bcl-2 | 3498 | 1:1000 | Cell Signaling Technology, USA |
| Bax | 5023 | 1:1000 | Cell Signaling Technology, USA |
| Bim | 2933 | 1:1000 | Cell Signaling Technology, USA |
| Cleaved PARP | 5625 | 1:1000 | Cell Signaling Technology, USA |
| Cleaved caspase 3 | 9661 | 1:1000 | Cell Signaling Technology, USA |
| Cleaved caspase 7 | 8438 | 1:1000 | Cell Signaling Technology, USA |
| NF-κB | 8242 | 1:1000 | Cell Signaling Technology, USA |
| Myd88 | 4283 | 1:1000 | Cell Signaling Technology, USA |
| Caspase 1 | 22915-1-AP | 1:1000 | Proteintech, Europe |
| NLRP3 | 15101 | 1:1000 | Cell Signaling Technology, USA |
| GSDMD | 69469 | 1:1000 | Cell Signaling Technology, USA |
| ZBP1 | 60968 | 1:1000 | Cell Signaling Technology, USA |
| MLKL | 37705 | 1:1000 | Cell Signaling Technology, USA |
| RIP1 | 3493 | 1:1000 | Cell Signaling Technology, USA |
| RIP3 | 10188 | 1:1000 | Cell Signaling Technology, USA |
